# National Assessment of Pediatric Readiness of US Emergency Departments During the COVID-19 Pandemic

**DOI:** 10.1001/jamanetworkopen.2023.21707

**Published:** 2023-07-07

**Authors:** Katherine E. Remick, Hilary A. Hewes, Michael Ely, Patricia Schmuhl, Rachel Crady, Lawrence J. Cook, Lorah Ludwig, Marianne Gausche-Hill

**Affiliations:** 1 Department of Pediatrics, Dell Medical School, University of Texas at Austin, Austin; 2 National Emergency Medical Services for Children Innovation and Improvement Center, Austin, Texas; 3 Emergency Medical Services (EMS) for Children Data Center, Salt Lake City, Utah; 4 University of Utah, Department of Pediatrics, Salt Lake City; 5 EMS for Children Branch, Maternal and Child Health Bureau, Health Resources and Services Administration, Department of Health and Human Services, Rockville, Maryland; 6 Departments of Emergency Medicine and Pediatrics, Harbor-UCLA Medical Center, Torrance, California; 7 The Lundquist Institute at Harbor-UCLA, Torrance, California; 8 Departments of Emergency Medicine and Pediatrics, David Geffen School of Medicine at UCLA, Los Angeles, California; 9 The Los Angeles County EMS Agency, Los Angeles, California

## Abstract

**Importance:**

The National Pediatric Readiness Project assessment provides a comprehensive evaluation of the readiness of US emergency departments (EDs) to care for children. Increased pediatric readiness has been shown to improve survival for children with critical illness and injury.

**Objectives:**

To complete a third assessment of pediatric readiness of US EDs during the COVID-19 pandemic, to examine changes in pediatric readiness from 2013 to 2021, and to evaluate factors associated with current pediatric readiness.

**Design, Setting, and Participants:**

In this survey study, a 92-question web-based open assessment of ED leadership in US hospitals (excluding EDs not open 24 h/d and 7 d/wk) was sent via email. Data were collected from May to August 2021.

**Main Outcomes and Measures:**

Weighted pediatric readiness score (WPRS) (range, 0-100, with higher scores indicating higher readiness); adjusted WPRS (ie, normalized to 100 points), calculated excluding points received for presence of a pediatric emergency care coordinator (PECC) and quality improvement (QI) plan.

**Results:**

Of the 5150 assessments sent to ED leadership, 3647 (70.8%) responded, representing 14.1 million annual pediatric ED visits. A total of 3557 responses (97.5%) contained all scored items and were included in the analysis. The majority of EDs (2895 [81.4%]) treated fewer than 10 children per day. The median (IQR) WPRS was 69.5 (59.0-84.0). Comparing common data elements from the 2013 and 2021 NPRP assessments demonstrated a reduction in median WPRS (72.1 vs 70.5), yet improvements across all domains of readiness were noted except in the administration and coordination domain (ie, PECCs), which significantly decreased. The presence of both PECCs was associated with a higher adjusted median (IQR) WPRS (90.5 [81.4-96.4]) compared with no PECC (74.2 [66.2-82.5]) across all pediatric volume categories (*P* < .001). Other factors associated with higher pediatric readiness included a full pediatric QI plan vs no plan (adjusted median [IQR] WPRS: 89.8 [76.9-96.7] vs 65.1 [57.7-72.8]; *P* < .001) and staffing with board-certified emergency medicine and/or pediatric emergency medicine physicians vs none (median [IQR] WPRS: 71.5 [61.0-85.1] vs 62.0 [54.3-76.0; *P* < .001).

**Conclusions and Relevance:**

These data demonstrate improvements in key domains of pediatric readiness despite losses in the health care workforce, including PECCs, during the COVID-19 pandemic, and suggest organizational changes in EDs to maintain pediatric readiness.

## Key Points

**Question:**

What is the state of pediatric readiness in US emergency departments (EDs) during the COVID-19 pandemic?

**Findings:**

In this survey study using a web-based assessment of leadership of 5150 EDs, 3647 (70.8%) responded, with a median weighted pediatric readiness score of 69.5 of 100. The presence of pediatric emergency care coordinators (PECCs), quality improvement plans, and board-certified emergency medicine or pediatric emergency medicine physicians were associated with better pediatric readiness; additionally, improvements have occurred in multiple domains since 2013 despite a reduction in the proportion of PECCs.

**Meaning:**

These findings suggest that pediatric readiness of US EDs was affected by the COVID-19 pandemic, yet engagement in these efforts remains strong.

## Teaser

This survey study presents results from an assessment of the pediatric readiness of US emergency departments during the COVID-19 pandemic and evaluates factors associated with current pediatric readiness.

## Introduction

Children represent approximately 25% of all emergency department (ED) visits, yet prior studies have highlighted variable readiness of EDs to meet the needs of children.^1^ The National Pediatric Readiness Project (NPRP) is a multiphase, multidisciplinary, longitudinal quality initiative to improve readiness of US EDs to care for children.^2^ NPRP is supported by the Health Resources and Services Administration/Emergency Medical Services (EMS) for Children Program and is cosponsored by the American Academy of Pediatrics (AAP), the American College of Emergency Physicians (ACEP), and the Emergency Nurses Association (ENA). This initiative has grown significantly over the last 2 decades since the first joint guidelines for care of children in the ED were published and with subsequent publication of revised guidelines in 2009 and 2018.^3,4,5,6,7,8,9,10,11^

Pediatric readiness efforts have included 2 previous national assessments of US EDs^12,13^ and multiple quality improvement (QI) collaboratives engaging more than 1000 ED personnel.^14^ Following the 2013 NPRP assessment of EDs, a large registry encompassing 83% of EDs became available for researchers to evaluate the association of pediatric readiness with patient outcomes.^15^ Ames et al^16^ found that EDs in the highest quartile of pediatric readiness had one-fourth the adjusted mortality for children with critical illness compared with EDs in the lowest quartile. Newgard et al^17^ found that injured children treated in the highest quartile EDs of 832 trauma centers (TCs) had approximately half the mortality rate of injured children treated in the lowest quartile EDs.^17^ A subsequent analysis of 983 EDs in 11 states found 60% lower mortality among injured children and 76% lower mortality among ill children in EDs with high pediatric readiness compared with those with low readiness and that these findings persisted up to 1 year following the ED visit.^18^ The objectives of the present study were to complete a third assessment of pediatric readiness of US EDs during the COVID-19 pandemic, to examine changes in pediatric readiness from 2013 to 2021, and to evaluate factors associated with current pediatric readiness.

## Methods

### Design, Setting, and Participants

In this survey study, a 92-question web-based open assessment of criteria identified in the 2018 AAP, ENA, ACEP joint guidelines was developed by the NPRP Steering Committee and was consistent with the American Association for Public Opinion Research (AAPOR) reporting.^9,10,11,19,20^ The NPRP assessment was reviewed and approved by the institutional review board at the University of Utah, Salt Lake City. Participation in the assessment was voluntary and informed consent was waived.^21^ Similar to the 2013 methods,^13^ a subpanel of experts developed weighting criteria, standardizing a formula for a weighted pediatric readiness score (WPRS) on a scale of 0 to 100 points, with higher values indicating higher readiness. The overall weighting of each domain of pediatric readiness remained consistent with the 2013 assessment (eAppendix 1 in Supplement 1).

ED demographics, including inpatient capabilities, ED configuration, trauma designation, and annual pediatric patient volume were collected. Field testing was conducted with 197 EDs in Colorado and Louisiana in the fall of 2020. Based on feedback, 4 assessment questions were modified before national deployment.

The assessment was deployed by the EMS for Children Data Center (EDC). EMS for Children (EMS-C) state partnership managers assisted with identification of ED contacts, most often a nurse manager.^22^ A total of 5150 EDs across 59 states and jurisdictions met inclusion criteria of accepting patients 24 h/d, 7 d/wk. Veterans Affairs and prison hospitals were excluded; military facilities were included if they otherwise met inclusion criteria. The assessment was open from May to August 2021.

Each ED contact was sent a prenotice, an initial invitation, and up to 4 reminders on a predetermined schedule to complete the assessment.^23^ Centralized outreach was supplemented by AAP, ACEP, and ENA awareness campaigns and EMS-C state partnership managers in each state and jurisdiction.^24,25^ Follow-up phone calls were made to nonresponding EDs by EDC staff and/or EMS-C state partnership managers. Upon completion of the assessment, each respondent received a gap report (eAppendix 2 in Supplement 1) of their overall WPRS and domain-specific results with links to the online NPRP toolkit.^26^ Only 1 response was allowed per ED. An interactive map of state and national response rates was publicly available^27^ (eAppendix 3 and eTable 1 in Supplement 1).

### Data Management

Online responses were collected in Checkbox survey software version 6.5 (Checkbox Technology). EDs were categorized by annual pediatric volume: low (<1800 patients), medium (1800-4999 patients), medium-high (5000-9999 patients), and high (≥10 000) volume. The median value within the selected volume category was used to estimate annual pediatric volume when the actual number of pediatric patient visits was unknown.

Hospital type included general hospital, children’s hospital within a general hospital, stand-alone children’s hospital, critical access hospital, microhospital, off-site hospital or satellite ED, independently owned freestanding ED, or other. The ED configuration was defined as a general ED (adults and children are seen in the same space), a separate pediatric ED in a hospital that treats both children and adults, a pediatric ED in a children’s hospital, or other. Hospital location was classified as urban, suburban, rural, or remote using the US Department of Agriculture’s 2013, 12-part, county Urban Influence Codes classification scheme.^28^ Emergency medicine and pediatric emergency medicine (EM/PEM) board certification was defined as physicians board-certified or eligible by the American Board of Emergency Medicine, the American Osteopathic Board of Emergency Medicine, and/or the American Board of Pediatrics in Pediatric Emergency Medicine.

### Statistical Analysis

ED demographic characteristics were summarized using frequencies and percentages for categorical data and medians for quantitative variables. Key components of the assessment, including overall score, were analyzed across pediatric patient volume categories. A Kruskal-Wallis test was used to evaluate median WPRS across EM board certification categories.

Overall, 69 of 88 (78.4%) scored items were comparable between the 2013 and 2021 assessments and were analyzed using a normalized score. To evaluate for improvement, a paired *t* test compared the overall WPRS and domain scores between the 2013 and 2021 cohorts and between those EDs that participated in both assessment periods.

To evaluate the presence of pediatric emergency care coordinators (PECCs) and QI plans on other elements within the assessment, each WPRS was adjusted by removing all points associated with having a PECC or QI plan and then dividing by the remaining points, 81 and 93 points, respectively. The adjusted WPRS (aWPRS) was compared across ED volumes using the Kruskal-Wallis test. A Kruskal-Wallis test was used to evaluate the distribution of median WPRS across EM/PEM board certification and the aWPRS for the QI plan; all are illustrated using boxplots. Odds ratios were calculated to determine the association of PECCs with readiness in each of the other domains.

All analyses were conducted in SAS software version 9.4 (SAS Institute). An α = .05 was used to determine statistical significance; all tests were 2-tailed.

## Results

Of the 5150 invitations sent, 3647 EDs (70.8%) responded. In August 2021, an issue with the online survey software necessitated closure of the platform. A PDF version of the assessment was made available to all remaining nonrespondents. A total of 505 PDF responses were submitted. Of these, 172 were missing answers to at least 1 scored element; 82 were later completed in full. The remaining 90 PDF responses were missing scored data elements. A sensitivity analysis, where missing responses were removed from denominators, demonstrated no changes in the statistical significance or conclusions derived from the results when all surveys were analyzed. The 90 surveys with missing scored data were excluded, and all results presented are based on the remaining 3557 assessments (97.5%).

The majority of EDs (2895 [81.4%]) had low or medium pediatric volume and treated fewer than 10 children per day (eFigure in Supplement 1). Overall, these data represent 14.1 million pediatric visits to EDs. There was no association between geolocation and response to the 2021 assessment (eTable 2 in Supplement 1).

The number of responses by ED location, hospital type, and ED configuration by pediatric patient volume category are summarized in Table 1. The majority of EDs were located in general hospitals (2108 [59.3%]) and urban areas (2239 [62.9%]). General EDs accounted for 90.4% of all EDs (3217). In addition, 97.8% of EDs (3477) were in non–stand-alone children’s hospitals and provided emergency care for an estimated 78.8% of children.

**Table 1.** Hospital and ED Characteristics

Characteristic	EDs, No. (%)				
	Pediatric patient volume category^a^				Overall (N = 3557)
	Low (n = 1793)	Medium (n = 1102)	Medium-high (n = 376)	High (n = 286)	
Urbanicity					
Urban	824 (46.0)	792 (71.9)	339 (90.2)	284 (99.3)	2239 (62.9)
Suburban	135 (7.5)	143 (13.0)	22 (5.9)	0	300 (8.4)
Rural	516 (28.8)	145 (13.2)	12 (3.2)	1 (0.3)	674 (18.9)
Remote	318 (17.7)	22 (2.0)	3 (0.8)	1 (0.3)	344 (9.7)
Hospital configuration					
General hospital	780 (43.5)	899 (81.6)	326 (86.7)	103 (36.0)	2108 (59.3)
Children’s hospital within a general hospital	1 (0.1)	9 (0.8)	32 (8.5)	94 (32.9)	136 (3.8)
Children’s hospital	0	0	3 (0.8)	77 (26.9)	80 (2.2)
Critical access	831 (46.3)	98 (8.9)	4 (1.1)	0	933 (26.2)
Microhospital	38 (2.1)	9 (0.8)	0	0	47 (1.3)
Satellite ED	86 (4.8)	65 (5.9)	10 (2.7)	6 (2.1)	167 (4.7)
Freestanding ED	20 (1.1)	11 (1.0)	0	0	31 (0.9)
Other	36 (2.0)	11 (1.0)	1 (0.3)	6 (2.1)	54 (1.5)
Missing	1 (0.1)	0	0	0	1 (0.0)
ED configuration					
General ED	1772 (98.8)	1063 (96.5)	321 (85.4)	61 (21.3)	3217 (90.4)
Separate pediatric ED	9 (0.5)	32 (2.9)	49 (13.0)	132 (46.2)	222 (6.2)
Pediatric ED	3 (0.2)	0	2 (0.5)	89 (31.1)	94 (2.6)
Other	8 (0.4)	7 (0.6)	3 (0.8)	4 (1.4)	22 (0.6)
Missing	1 (0.1)	0	1 (0.3)	0	2 (0.1)
Pediatric inpatient capability					
Admits children to pediatric-specific unit^b^	253 (14.1)	371 (33.7)	225 (59.8)	254 (88.8)	1103 (31.0)
Admits children to adult unit and newborns	346 (19.3)	232 (21.1)	47 (12.5)	8 (2.8)	633 (17.8)
Admits only newborns	152 (8.5)	208 (18.9)	65 (17.3)	10 (3.5)	435 (12.2)
Admits children to adult unit only	468 (26.1)	74 (6.7)	7 (1.9)	1 (0.3)	550 (15.5)
Does not admit children	573 (32.0)	217 (19.7)	32 (8.5)	13 (4.5)	835 (23.5)
Missing	1 (0.1)	0	0	0	1 (<0.1)

Abbreviation: ED, emergency department.

^a^ Pediatric patient volume ranges: low (<1800), medium (1800-4999), medium-high (4999-9999), and high (>10 000) volume.

^b^ Includes pediatric ward, step-down or intermediate care units, and/or pediatric intensive care units with or without neonatal-specific services.

Among all respondents, 2722 (76.5%) admitted children in some capacity, and 1737 (48.8%) admitted children to adult inpatient units. The majority offered neonatal inpatient services (2058 [57.9%]), fewer offered pediatric inpatient ward services (1098 [30.9%]), and 344 (9.7%) had a dedicated pediatric intensive care unit (PICU).

Less than half of respondents reported their hospital is a designated TC (1595 [44.8%]). Of designated TCs, 1002 (62.8%) were verified by a state or regional entity only, 361 (22.6%) by the American College of Surgeons (ACS) only, 227 (14.2%) by both ACS and a state or regional entity, and 5 (0.3%) did not report type of verification. Only 212 (13.3%) were pediatric TCs.

### ED Staffing

Among respondents, 3215 (90.4%) reported a physician on site 24 h/d and 7 d/wk. Overall, 90% of EDs required physician staff that care for children be board certified or eligible, with the large majority (2704 [84.1%]) requiring EM certification and 454 (14.1%) requiring PEM (Table 2). More than 43% of EDs (1411) were staffed with only EM/PEM–boarded physicians.

**Table 2.** National Pediatric Readiness Project Assessment Response Summary

Characteristic	EDs, No. (%)				
	Pediatric patient volume category^a^				Overall (n = 3557)
	Low (n = 1793)	Medium (n = 1102)	Medium high (n = 376)	High (n = 286)	
WPRS, median (IQR)^b^	64.0 (55.6-76.0)	71.4 (61.0-85.4)	77.5 (66.1-91.0)	94.4 (83.3-97.5)	69.5 (59.0- 84.0)
PECC					
Physician PECC					
MD or DO	432 (24.1)	425 (38.6)	197 (52.4)	225 (78.7)	1279 (36.0)
Advanced practice practitioner	32 (1.8)	10 (0.9)	1 (0.3)	3 (1.0)	46 (1.3)
Nurse PECC					
RN	502 (28.0)	426 (38.7)	182 (48.4)	210 (73.4)	1320 (37.1)
Advanced practice practitioner	2 (0.1)	5 (0.5)	0	5 (1.7)	12 (0.3)
Physician certifications and training (board)^c^					
Emergency medicine board eligible or certified	1141 (77.8)	990 (90.6)	347 (93.3)	226 (79.6)	2704 (84.1)
Pediatric emergency medicine board eligible or certified	78 (5.3)	100 (9.1)	62 (16.7)	214 (75.4)	454 (14.1)
Pediatrics board eligible or certified	77 (5.3)	83 (7.6)	64 (17.2)	182 (64.1)	406 (12.6)
Family medicine board eligible or certified	536 (36.6)	308 (28.2)	73 (19.6)	31 (10.9)	948 (29.5)
Other eligible or certified	551 (37.6)	354 (32.4)	99 (26.6)	60 (21.1)	1064 (33.1)
Non–board eligible or certified physician with other training	207 (14.1)	103 (9.4)	22 (5.9)	19 (6.7)	351 (10.9)
ED competency evaluations					
Physician	1111 (62.0)	750 (68.1)	277 (73.7)	257 (89.9)	2395 (67.3)
Nurse	1536 (85.7)	997 (90.5)	360 (95.7)	272 (95.1)	3165 (89.0)
Advanced practice practitioner^d^	681 (67.4)	595 (66.0)	212 (66.3)	192 (85.3)	1680 (68.4)
Pediatric-specific policies or procedures					
QI process	738 (41.2)	564 (51.2)	222 (59.0)	253 (88.5)	1777 (50.0)
Weight in kilograms	1177 (65.6)	873 (79.2)	333 (88.6)	268 (93.7)	2651 (74.5)
Triage	934 (52.1)	731 (66.3)	290 (77.1)	263 (92.0)	2218 (62.4)
Patient assessment and reassessment	1303 (72.7)	905 (82.1)	321 (85.4)	271 (94.8)	2800 (78.7)
Immunization assessment and management	702 (39.2)	532 (48.3)	188 (50.0)	204 (71.3)	1626 (45.7)
Child maltreatment	1573 (87.7)	1021 (92.6)	359 (95.5)	277 (96.9)	3230 (90.8)
Death in ED	1137 (63.4)	835 (75.8)	283 (75.3)	269 (94.1)	2524 (71.0)
Reduced-dose radiation for CT and radiograph imaging	1261 (70.3)	864 (78.4)	305 (81.1)	271 (94.8)	2701 (75.9)
Mental health care	1155 (64.4)	877 (79.6)	297 (79.0)	270 (94.4)	2599 (73.1)
Behavioral health transfer	1051 (58.6)	790 (71.7)	268 (71.3)	255 (89.2)	2364 (66.5)
Social service plans	1003 (55.9)	811 (73.6)	310 (82.4)	265 (92.7)	2389 (67.2)
Interfacility guidelines for transfer of pediatric patients	1187 (66.2)	818 (74.2)	300 (79.8)	245 (85.7)	2550 (71.7)
Family-centered care plan	1002 (55.9)	716 (65.0)	262 (69.7)	244 (85.3)	2224 (62.5)
Disaster planning	676 (37.7)	546 (49.5)	231 (61.4)	238 (83.2)	1691 (47.5)
Percentage of recommended equipment carried^e^					
Median (IQR)	100.0 (95.3-100.0)	100.0 (97.7-100.0)	100.0 (97.7-100.0)	100.0 (100.0-100.0)	100.0 (95.3-100.0)
100% of recommended equipment carried	904 (50.4)	707 (64.2)	249 (66.2)	245 (85.7)	2105 (59.2)

Abbreviations: CT, computed tomography; ED, emergency department; PECC, pediatric emergency care coordinator; QI, quality improvement; WPRS, weighted pediatric readiness score.

^a^ Pediatric patient volume ranges: low (<1800), medium (1800-4999), medium-high (4999-9999), and high (>10 000) volume.

^b^ Kruskal-Wallis Test of WPRS against volume, *P* < .001.

^c^ Calculation based on EDs that have a physician working onsite 24 h/d and 7 d/wk; missingness in board certification includes: emergency medicine (20), pediatric emergency medicine (72), pediatrics (78), family medicine (71), other certification (71), and non–board eligible (83).

^d^ Calculation based on EDs that employ advanced practice practitioners.

^e^ Overall, 92% of EDs had at least 90% of equipment.

### Pediatric Readiness Score

The overall median (IQR) WPRS was 69.5 (59.0-84.0). The score increased with pediatric patient volume (*P* < .001) (Table 2).

### Administration and Coordination

The presence of a physician PECC was reported by 1325 EDs (37.3%), a nurse PECC in 1332 (37.4%), and each varied by pediatric volume (Table 2). Dedicated nonclinical time to complete responsibilities was reported for 1013 physician PECCs (76.5%) and 1081 nurse PECCs (81.2%). There were 1014 EDs (28.5%) that reported having both a physician and nurse PECC.

### Physicians and Nurse Competencies

Pediatric-specific competencies for credentialing were required for physicians (2395 [67.3%]) and nurses (3165 [89.0%]). Of the 2457 EDs (69.1%) that employed advanced practice practitioners (APPs), 1680 (68.4%) required competency evaluations (Table 2).

### QI Plan

Among respondents, 1777 (50.0%) reported having a pediatric inclusive QI plan (Table 2). Of those, 1069 (60.2%) had all QI elements, including pediatric quality indicators and reevaluation of performance using outcome-based measures.

### Patient Safety

Vital sign measurement for children (eg, pulse oximetry, vital signs recorded, and blood pressure monitoring) was available in more than 99% of EDs (3523). Pediatric patient safety factors, such as a process for precalculated drug dosing (3036 [85.4%]) and weighing plus recording weights in kilograms only (2651 [74.5%]), were also largely present.

### Policies, Procedures, and Protocols

Most EDs maintain recommended policies, procedures, and protocols (Table 2). Among EDs with a family-centered care policy, 1533 (68.9%) had all recommended elements. Most respondents reported having interfacility transfer guidelines for ill and injured children (2550 [71.7%]), and 2433 (68.4%) had pediatric interfacility transfer agreements. Less than half of respondents reported the inclusion of pediatric components in the hospital disaster plan (1691 [47.5%]).

### Equipment and Supplies

Of respondents, more than 99% (3529) reported staff are trained on the location of pediatric equipment and medications in the ED and have a standardized tool to estimate weight during a resuscitation. Most (2992 [84.2%]) reported a daily method to verify the proper location and stocking of pediatric equipment and supplies. Overall, the median (IQR) percentage of recommended pediatric equipment was 100% (95.3%-100%), and 92% of EDs (3267) had more than 90% of the equipment. Commonly missing equipment included infant-sized nonrebreather masks (410 [11.5%]), pediatric-sized Magill forceps (408 [11.5%]), pediatric difficult airway kit (278 [7.8%]), and neonatal blood pressure cuffs (241 [6.8%]).

### Change in NPRP Elements Over Time

A comparison of 2013 and 2021 assessment demographics can be found in eTable 3 in Supplement 1. When comparing common scored data elements only, the overall median (IQR) WPRS decreased from 72.1 (59.5-86.4) in 2013 to 70.5 (61.6-87.3) in 2021 among all respondents (*P* = .13) (Table 3). Improvement from 2013 was notable in every domain except administration and coordination (ie, presence of a PECC), which declined most notably in low- and medium-volume EDs (eTable 4 in Supplement 1).

**Table 3.** Change in Total and Domain Scores Over Time Normalized for Common Scored Data Elements^a^

Element	Score, median (IQR)				*P* value^b^
	2013, All hospitals (n = 4146)	2021, All hospitals (n = 3557)	2013, Both years (n = 2825)	2021, Both years (n = 2825)	
WPRS	72.2 (59.7-86.5)	70.5 (61.6-87.3)	72.7 (60.1-87.8)	70.5 (61.3-87.4)	.13
Administration and coordination domain score	9.5 (0.0-19.0)	0.0 (0.0-19.0)	9.5 (0.0-19.0)	0.0 (0.0-19.0)	<.001
Personnel domain score	5.0 (0.0-10.0)	10.0 (5.0-10.0)	5.0 (0.0-10.0)	10.0 (5.0-10.0)	<.001
Quality improvement domain score	0.0 (0.0-5.6)	0.0 (0.0-7.0)	0.0 (0.0-5.6)	0.0 (0.0-7.0)	.001
Patient safety domain score	12.1 (10.2-14.0)	14.0 (12.1-14.0)	12.1 (10.2-14.0)	14.0 (12.1-14.0)	<.001
Policies and procedures domain score	11.8 (7.4-14.8)	12.6 (8.9-17.0)	11.8 (7.4-14.8)	12.6 (8.9-17.0)	<.001
Equipment and supplies domain score	32.4 (30.1-33.0)	33.0 (31.3-33.0)	32.4 (30.1-33.0)	33.0 (31.3-33.0)	<.001

^a^ All scores normalized for common data elements; 78% of scored questions in the 2021 survey were the same in 2013.

^b^ *P* value calculated from Wilcoxon signed-rank test comparing domain scores across EDs that took the assessment both years (2825 EDs [79.4%]).

### Board Certification, QI Plans, PECCs, and Pediatric Readiness

Staffing the ED with board-certified or eligible EM or PEM physicians vs none was associated with significantly higher median (IQR) WPRS (71.5 [61.0-85.1] vs 62.0 [54.3-76.0]; *P* < .001) (Figure, A). Adjusting for point values for QI plans, the presence of an inclusive QI plan for children vs none was associated with a significant increase in overall median (IQR) aWPRS regardless of pediatric volume (89.8 [76.9-96.7] vs 65.1 [57.7-72.8]; *P* < .001) (Figure, B). Adjusting for the point values for PECCs, the presence of either a physician or nurse PECC vs none was associated with an overall increased median (IQR) WPRS regardless of pediatric volume, and this was even more pronounced when both a physician and a nurse PECC were identified (90.5 [81.4-96.4] vs 74.2 [66.2-82.5]; *P* < .001) (Figure, C). The presence of PECCs was associated with increased odds of having all remaining components of each pediatric readiness domain (*P* < .001) (Table 4).

**Figure.**
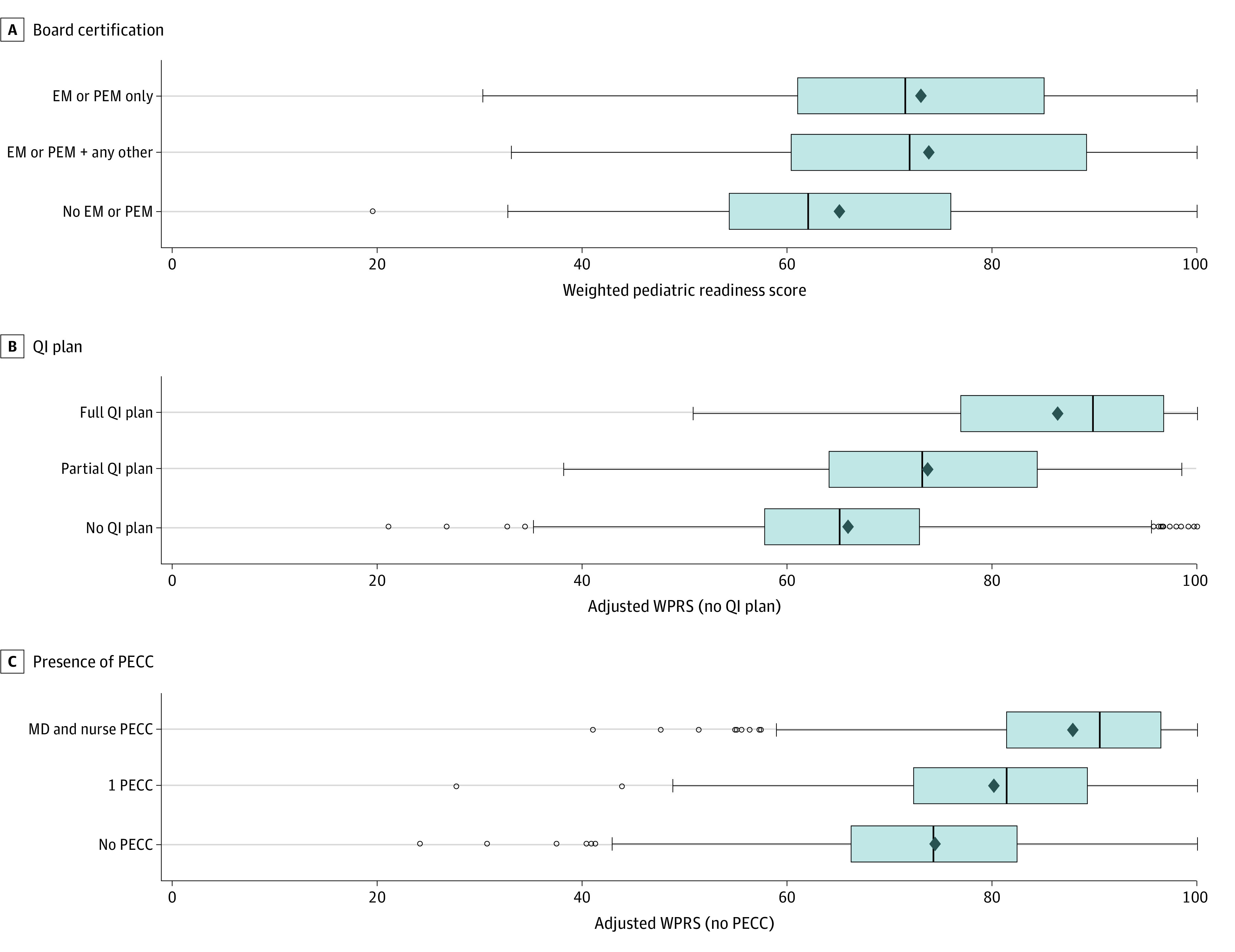
Weighted Pediatric Readiness Score by Emergency Medicine Board Certification, Presence of a Quality Improvement (QI) Plan, and Presence of a Pediatric Emergency Care Coordinator (PECC) A, Emergency department staffing includes board-certified or eligible emergency medicine (EM) or pediatric emergency physicians (PEM) (only EM or PEM), some staff EM or PEM, and no staff EM or PEM. B, Emergency department has a QI plan that has all the components as outlined in national guidelines for care of children (full QI plan); has some of the components of the recommended QI plan (partial QI plan); no QI plan for children (no QI plan). C, Emergency department has both a physician and nurse PECC (MD and nurse PECC); has either a nurse or physician PECC (1 PECC); or has neither a physician nor a nurse PECC (No PECCs).

**Table 4.** Odds of Perfect Domain Score by PECC Presence^a^

Domains of pediatric readiness	No PECC (n = 1914)	≥1 PECC (physician, nurse, or both) (n = 1643)	Odds ratio (95% CI)	*P* value
Equipment and supplies (33 of 33 points)	864 (45.1)	999 (60.8)	1.89 (1.65-2.16)	<.001
Patient safety (14 of 14 points)	900 (47.0)	1091 (66.4)	2.23 (1.94-2.55)	<.001
Personnel training and competencies (10 of 10 points)	166 (8.7)	336 (20.5)	2.71 (2.22-3.31)	<.001
Policies and procedures (17 of 17 points)	140 (7.3)	351 (21.4)	3.44 (2.80-4.25)	<.001
Quality improvement plan (7 of 7 points)	249 (13.0)	820 (49.9)	6.66 (5.66-7.87)	<.001

Abbreviation: PECC, pediatric emergency care coordinator.

^a^ Results are based on univariable model(s).

## Discussion

We report on a web-based assessment of all US EDs for incorporation of the national guidelines for pediatric readiness. The 70.8% response rate, and an overall readiness score of 69.5, provides a comprehensive snapshot of the state of pediatric readiness in EDs during the COVID-19 pandemic. The pediatric readiness score did not demonstrate significant overall improvement since 2013, however there remains a high level of engagement by frontline health care professionals in pediatric readiness efforts, as noted by improvements in all domains of readiness except administration and coordination.

Although other investigators, who surveyed ED directors using a smartphone mobile application, found that less than 20% of EDs have assigned the role of a PECC, we report that 37.3% of responders assigned a physician PECC and 37.4% a nurse PECC.^29^ Compared with previous reports that found 48% of responders reported a physician PECC and 59% a nurse PECC, the number of PECCs has significantly decreased.^13^ This reduction in PECCs likely reflects pandemic changes in workforce deployment from administrative functions to clinical care or being furloughed.^30,31^ The decrease in PECCs suggests a vulnerability of pediatric readiness coordination within EDs and a potential gap in the ability of EDs to address deficiencies in pediatric emergency care, most pronounced in smaller community EDs.

Prior to the COVID-19 pandemic, reports estimated 35 million annual pediatric ED visits in the US.^32,33^ In 2020, that number decreased by more than 50%, which is similarly reflected in this respondent cohort representing approximately 14 million pediatric visits.^34^ The decrease in pediatric volume does not necessarily equate to a decrease in severity or need, as many children delayed seeking care, resulting in increased morbidity for illness or injury.^35,36,37,38,39,40^ The availability of pediatric inpatient services also declined since the 2013 NPRP assessment, including a decrease in hospitals reporting PICU beds (12.5% to 9.7%) and pediatric inpatient ward beds (53.4% to 30.8%). Hence, the burden of inpatient pediatric care is shifting to regional pediatric hospitals, which results in greater numbers of transfers and patients being cared for outside their communities, limiting, for some, access to pediatric medical care.^41,42,43^ Thus, the loss of pediatric inpatient availability makes ED stabilization through optimized pediatric readiness even more relevant.

This report underscores the importance of QI plans that include children and staffing the ED with board-certified emergency physicians. These elements of pediatric readiness drive quality within the ED, which ultimately improves the overall responsiveness of ED staff to care for children with critical illness and injury.^44^

A number of important improvements in EDs as well as additional opportunities for QI are noted in this report. Weighing and recording pediatric patients in kilograms has been an important safety initiative to ensure appropriate dosing of all medications.^45,46,47^ In previous reports, less than two-thirds of respondents reported weighing and recording in kilograms only, which has increased to 74.5% in this report. We report an increase in the recommended equipment and supplies for children stocked in EDs, with 59% stocking 100% of the recommended equipment, up from 20% in previous reports.^13^ Finally, only 62.4% of all EDs reported the presence of a triage policy that addresses children although the ENA endorsed the Emergency Severity Index triage tool^48^ as a validated method to assess the criticality of pediatric patients.

A growing awareness of potential threats in virtually every community and the low percentage of hospitals (48%) that include children as a part of their hospital disaster plan highlight the importance of hospitals working collaboratively within regional health care coalitions to ensure the US emergency care system is resilient and has the capacity and capability to meet the day-to-day and disaster-imposed needs of children. An important step toward building this resilience was the American College of Surgeons adopting pediatric readiness requirements for all verified TCs.^49,50^ This engagement by national professional organizations as well as by federal agencies in pediatric readiness efforts is impactful and has significant public health implications.

### Limitations

This study has limitations. These data were self-reported by a single individual representing each ED in a leadership role and may not represent universal knowledge or awareness of available pediatric resources among all ED staff. Despite a high national response rate, missing scored data elements led to the exclusion of 2.5% of responses (90). We have limited data on nonrespondents that can potentially introduce bias and affect the results reported. Furthermore, the number of reported EDs in each state was determined by the EMS-C program managers and is subject to reporting bias.

## Conclusions

These data represent an updated report of the state of pediatric readiness in our nation’s EDs during a global pandemic and demonstrate high engagement of ED leadership. During the 8 years since the last national assessment, all domains of readiness have increased except administration in the ED (ie, PECCs), which declined significantly. Although the role of the PECC is central to improving pediatric readiness, stressors on the health care sector and its workforce have undoubtedly played a role in reducing the assignment of physician and nurse personnel to this role. This comprehensive assessment found that the presence of PECCs, QI plans for children, and staffing the ED with board-certified EM/PEM physicians were associated with higher pediatric readiness and provides an opportunity for all EDs to initiate organizational changes that can enhance their pediatric capability.

## Notes

Supplement 1. **eAppendix 1.** National Pediatric Readiness Project (NPRP) Assessment With Weighted Elements **eAppendix 2.** National Pediatric Readiness Project Sample (NPRP) Gap Report **eAppendix 3.** National Pediatric Readiness Project (NPRP) Response Rate Map **eFigure.** Emergency Department Volume Category and Pediatric Visits per Day **eTable 1.** National Pediatric Readiness Project (NPRP) Response Rate Table by State **eTable 2.** Comparison of Nonrespondents With Respondents of the 2021 National Pediatric Readiness Project (NPRP) Assessment **eTable 3.** Comparison of Site Demographics: 2013-2021 National Pediatric Readiness Project (NPRP) Assessments **eTable 4. **Comparison of Domain Scores by Pediatric Volume Categories: 2013-2021 National Pediatric Readiness Project (NPRP) Assessments

Supplement 2.
Data Sharing Statement

## References

[1] Institute of Medicine; Committee of the Future of Emergency Care in the U.S. Health System. Emergency Care for Children: Growing Pains. National Academy Press; 2006.

[2] EMS for Children Innovation and Improvement Center. National Pediatric Readiness Project. Accessed October 30, 2022. https://emscimprovement.center/domains/pediatric-readiness-project/about

[3] Remick K, Snow S, Gausche-Hill M. Emergency department readiness for pediatric illness and injury. Pediatr Emerg Med Pract. 2013;10(12):1-13. 24494276

[4] American Academy of Pediatrics, Committee on Pediatric Emergency Medicine and American College of Emergency Physicians, and Pediatric Committee. Care of children in the emergency department: guidelines for preparedness. Pediatrics. 2001;107(4):777-781. doi:10.1542/peds.107.4.777 11335759

[5] American College of Emergency Physicians; American Academy of Pediatrics. Care of children in the emergency department: guidelines for preparedness. Ann Emerg Med. 2001;37(4):423-427. doi:10.1067/mem.2001.114067 11275847

[6] American Academy of Pediatrics, Committee on Pediatric Emergency Medicine; American College of Emergency Physicians, Pediatric Committee; Emergency Nurses Association, Pediatric Committee. Joint policy statement—guidelines for care of children in the emergency department. J Emerg Nurs. 2013;39(2):116-131. doi:10.1016/j.jen.2013.01.003 23498882

[7] American Academy of Pediatrics; Committee on Pediatric Emergency Medicine; American College of Emergency Physicians; Pediatric Committee; Emergency Nurses Association Pediatric Committee. Joint policy statement—guidelines for care of children in the emergency department. Pediatrics. 2009;124(4):1233-1243. doi:10.1542/peds.2009-1807 19770172

[8] American Academy of Pediatrics Committee on Pediatric Emergency Medicine; American College of Emergency Physicians Pediatric Committee; Emergency Nurses Association Pediatric Committee. Joint policy statement—guidelines for care of children in the emergency department. Ann Emerg Med. 2009;54(4):543-552. doi:10.1016/j.annemergmed.2009.08.010 19769888

[9] Remick K, Gausche-Hill M, Joseph MM, Brown K, Snow SK, Wright JL; AMERICAN ACADEMY OF PEDIATRICS Committee on Pediatric Emergency Medicine and Section on Surgery; AMERICAN COLLEGE OF EMERGENCY PHYSICIANS Pediatric Emergency Medicine Committee; EMERGENCY NURSES ASSOCIATION Pediatric Committee. Pediatric readiness in the emergency department. Pediatrics. 2018;142(5):e20182459. doi:10.1542/peds.2018-2459 30819971

[10] Remick K, Gausche-Hill M, Joseph MM, Brown K, Snow SK, Wright JL; AMERICAN ACADEMY OF PEDIATRICS, Committee on Pediatric Emergency Medicine, Section on Surgery; AMERICAN COLLEGE OF EMERGENCY PHYSICIANS, Pediatric Emergency Medicine Committee; EMERGENCY NURSES ASSOCIATION, Pediatric Committee; Pediatric Readiness in the Emergency Department; POLICY STATEMENT; Organizational Principles to Guide and Define the Child Health Care System and/or Improve the Health of All Children. Pediatric readiness in the emergency department. Ann Emerg Med. 2018;72(6):e123-e136. doi:10.1016/j.annemergmed.2018.08.431 30392738

[11] Remick K, Gausche-Hill M, Joseph MM, Brown K, Snow SK, Wright JL; AMERICAN ACADEMY OF PEDIATRICS, Committee on Pediatric Emergency Medicine, Section on Surgery; AMERICAN COLLEGE OF EMERGENCY PHYSICIANS, Pediatric Emergency Medicine Committee; EMERGENCY NURSES ASSOCIATION, Pediatric Committee; Pediatric Readiness in the Emergency Department; POLICY STATEMENT; Organizational Principles to Guide and Define the Child Health Care System and/or Improve the Health of All Children. Pediatric readiness in the emergency department. J Emerg Nurs. 2019;45(1):e3-e18. doi:10.1016/j.jen.2018.10.003 30392719

[12] Gausche-Hill M, Schmitz C, Lewis RJ. Pediatric preparedness of US emergency departments: a 2003 survey. Pediatrics. 2007;120(6):1229-1237. doi:10.1542/peds.2006-3780 18055671

[13] Gausche-Hill M, Ely M, Schmuhl P, . A national assessment of pediatric readiness of emergency departments. JAMA Pediatr. 2015;169(6):527-534. doi:10.1001/jamapediatrics.2015.138 25867088

[14] EMS for Children Innovation and Improvement Center. Quality collaboratives. Accessed October 30, 2022. https://emscimprovement.center/collaboratives/all

[15] National EMS for Children Data Analysis Resource Center. Pediatric readiness research opportunities. Accessed December 20, 2022. https://www.nedarc.org/pedsReady/pedsReadyResearch.html

[16] Ames SG, Davis BS, Marin JR, . Emergency department pediatric readiness and mortality in critically ill children. Pediatrics. 2019;144(3):e20190568. doi:10.1542/peds.2019-0568 31444254 PMC6856787

[17] Newgard CD, Lin A, Olson LM, ; Pediatric Readiness Study Group. Evaluation of emergency department pediatric readiness and outcomes among US trauma centers. JAMA Pediatr. 2021;175(9):947-956. doi:10.1001/jamapediatrics.2021.1319 34096991 PMC8185631

[18] Newgard CD, Lin A, Malveau S, ; Pediatric Readiness Study Group. Emergency department pediatric readiness and short-term and long-term mortality among children receiving emergency care. JAMA Netw Open. 2023;6(1):e2250941. doi:10.1001/jamanetworkopen.2022.50941 36637819 PMC9857584

[19] Eysenbach G. Improving the quality of web surveys: the Checklist for Reporting Results of Internet E-Surveys (CHERRIES). J Med Internet Res. 2004;6(3):e34. doi:10.2196/jmir.6.3.e34 15471760 PMC1550605

[20] American Association for Public Opinion Research (AAPOR). Best Practices for Research. Accessed April 27, 2023. https://aapor.org/standards-and-ethics/best-practices#1668112078364-fc92d558-e761

[21] National Pediatric Readiness Project. Accessed October 30, 2022. https://www.pedsready.org

[22] EMS for Children Innovation and Improvement Center. State partnerships grants. Accessed October 30, 2022. https://emscimprovement.center/programs/partnerships

[23] Dillman DA, Smyth JD, Christian LM. Internet, Mail, and Mixed-Mode Surveys: The Tailored Design Method. John Wiley & Sons, Inc; 2014.

[24] EMS for Children Innovation and Improvement Center. Two webinars to be held on pediatric readiness. Accessed October 30, 2022. https://emscimprovement.center/news/two-webinars-be-held-pediatric-readiness

[25] EMS for Children Innovation and Improvement Center. State program managers. Accessed October 30, 2022. https://emscimprovement.center/programs/grants

[26] EMS for Children Innovation and Improvement Center. ED Readiness Toolkit. Accessed October 30, 2022. https://emscimprovement.center/domains/pediatric-readiness-project/readiness-toolkit/readiness-toolkit-checklist

[27] National Pediatric Readiness Project. 2021 National Pediatric Readiness Assessment response rates. Accessed October 30, 2021. https://tinyurl.com/PedsReadyRR

[28] US Department of Agriculture. Urban Influence Codes. Accessed November 2, 2022. https://www.ers.usda.gov/data-products/urban-influence-codes/urban-influence-codes

[29] Boggs KM, Espinola JA, Sullivan AF, . Availability of pediatric emergency care coordinators in United States emergency departments. J Pediatr. 2021;235(235):163-169.e1. doi:10.1016/j.jpeds.2021.02.014 33577802

[30] Assistant Secretary for Planning and Evaluation. Impact of the COVID-19 pandemic on the hospital and outpatient clinician workforce. May 3, 2022. Accessed January 6, 2023. https://aspe.hhs.gov/sites/default/files/documents/9cc72124abd9ea25d58a22c7692dccb6/aspe-covid-workforce-report.pdf

[31] Paavola A. 266 Hospitals furloughing workers in response to COVID-19. Beckers Hospital Review. Accessed December 28, 2022. https://www.beckershospitalreview.com/finance/49-hospitals-furloughing-workers-in-response-to-covid-19.html

[32] Whitfill T, Auerbach M, Scherzer DJ, Shi J, Xiang H, Stanley RM. Emergency care for children in the United States: epidemiology and trends over time. J Emerg Med. 2018;55(3):423-434. doi:10.1016/j.jemermed.2018.04.019 29793812

[33] Radhakrishnan L, Carey K, Hartnett KP, . Pediatric emergency department visits before and during the COVID-19 pandemic—United States, January 2019-January 2022. MMWR Morb Mortal Wkly Rep. 2022;71(8):313-318. doi:10.15585/mmwr.mm7108e1 35202351

[34] Hartnett KP, Kite-Powell A, DeVies J, ; National Syndromic Surveillance Program Community of Practice. Impact of the COVID-19 pandemic on emergency department visits—United States, January 1, 2019-May 30, 2020. MMWR Morb Mortal Wkly Rep. 2020;69(23):699-704. doi:10.15585/mmwr.mm6923e1 32525856 PMC7315789

[35] Leeb RT, Bitsko RH, Radhakrishnan L, Martinez P, Njai R, Holland KM. Mental health–related emergency department visits among children aged <18 years during the COVID-19 Pandemic—United States, January 1-October 17, 2020. MMWR Morb Mortal Wkly Rep. 2020;69(45):1675-1680. doi:10.15585/mmwr.mm6945a3 33180751 PMC7660659

[36] Swedo E, Idaikkadar N, Leemis R, . Trends in U.S. emergency department visits related to suspected or confirmed child abuse and neglect among children and adolescents aged <18 years before and during the COVID-19 pandemic—United States, January 2019-September 2020. MMWR Morb Mortal Wkly Rep. 2020;69(49):1841-1847. doi:10.15585/mmwr.mm6949a1 33301436 PMC7737689

[37] Metcalf S, Marlow JA, Rood CJ, Hilado MA, DeRidder CA, Quas JA. Identification and incidence of child maltreatment during the COVID-19 pandemic. Psychol Public Policy Law. 2022;28(2):267-279. doi:10.1037/law0000352 37206908 PMC10195111

[38] Gerall CD, DeFazio JR, Kahan AM, . Delayed presentation and sub-optimal outcomes of pediatric patients with acute appendicitis during the COVID-19 pandemic. J Pediatr Surg. 2021;56(5):905-910. doi:10.1016/j.jpedsurg.2020.10.008 33220973 PMC7569380

[39] Ciacchini B, Tonioli F, Marciano C, . Reluctance to seek pediatric care during the COVID-19 pandemic and the risks of delayed diagnosis. Ital J Pediatr. 2020;46(1):87. doi:10.1186/s13052-020-00849-w 32600464 PMC7322712

[40] Jain A, Patel A, Oyoyo U, Wiafe S. Impact of COVID 19 pandemic on health care utilization for patients with sickle cell disease—a specialty treatment center experience. Blood. 2021;138(Supplement 1):4955. doi:10.1182/blood-2021-151375

[41] Gausche-Hill M. Emergency and definitive care for children in the United States: the perfect storm. Pediatrics. 2020;145(1):e20193372. doi:10.1542/peds.2019-3372 31882441

[42] Michelson KA, Hudgins JD, Lyons TW, Monuteaux MC, Bachur RG, Finkelstein JA. Trends in capability of hospitals to provide definitive acute care for children: 2008 to 2016. Pediatrics. 2020;145(1):e20192203. doi:10.1542/peds.2019-2203 31882440

[43] Lieng MK, Marcin JP, Sigal IS, . Association between emergency department pediatric readiness and transfer of noninjured children in small rural hospitals. J Rural Health. 2022;38(1):293-302. doi:10.1111/jrh.12566 33734494 PMC8489899

[44] Brennan TA, Horwitz RI, Duffy FD, Cassel CK, Goode LD, Lipner RS. The role of physician specialty board certification status in the quality movement. JAMA. 2004;292(9):1038-1043. doi:10.1001/jama.292.9.1038 15339894

[45] Alessandrini E, Varadarajan K, Alpern ER, ; Pediatric Emergency Care Applied Research Network. Emergency department quality: an analysis of existing pediatric measures. Acad Emerg Med. 2011;18(5):519-526. doi:10.1111/j.1553-2712.2011.01057.x 21569170

[46] Alessandrini EA, Knapp J. Measuring quality in pediatric emergency care. Clin Pediatr Emerg Med. 2014;12:102-112. doi:10.1016/j.cpem.2011.05.002

[47] Remick KE, Bartley KA, Gonzales L, MacRae KS, Edgerton EA. Consensus-driven model to establish paediatric emergency care measures for low-volume emergency departments. BMJ Open Qual. 2022;11(3):e001803. doi:10.1136/bmjoq-2021-001803 35803615 PMC9272131

[48] Emergency Nurses Association. Triage offerings. Accessed December 20, 2033. https://www.ena.org/enau/educational-offerings/triage

[49] US Department of Health and Human Services. Pediatric surge resources. Accessed December 20, 2022. https://asprtracie.hhs.gov/pediatric-surge

[50] American College of Surgeon. Resources for optimal care of the injured patient. Accessed January 6, 2022. https://www.facs.org/quality-programs/trauma/quality/verification-review-and-consultation-program/standards/

