# Supplementary material for: National Assessment of Pediatric Readiness of US Emergency Departments During the COVID-19 Pandemic
Source: JAMA Netw Open. 2023 Jul 7;6(7):e2321707. doi: 10.1001/jamanetworkopen.2023.21707 (PMC10329204; doi:10.1001/jamanetworkopen.2023.21707)
Supplement: Supplement 1. — eAppendix 1. National Pediatric Readiness Project (NPRP) Assessment With Weighted Elements eAppendix 2. National Pediatric Readiness Project Sample (NPRP) Gap Report eAppendix 3. National Pediatric Readiness Project (NPRP) Response Rate Map eFigure. Emergency Department Volume Category and Pediatric Visits per Day eTable 1. National Pediatric Readiness Project (NPRP) Response Rate Table by State eTable 2. Comparison of Nonrespondents With Respondents of the 2021 National Pediatric Readiness Project (NPRP) Assessment eTable 3. Comparison of Site Demographics: 2013-2021 National Pediatric Readiness Project (NPRP) Assessments eTable 4. Comparison of Domain Scores by Pediatric Volume Categories: 2013-2021 National Pediatric Readiness Project (NPRP) Assessments [file jamanetwopen-e2321707-s001.pdf]

Supplemental Online Content

Remick KE, Hewes HA, Ely M, et al. National assessment of pediatric readiness of US emergency departments during the COVID-19 pandemic. *JAMA Netw Open.* 2023;6(7):e2321707. doi:10.1001/jamanetworkopen.2023.21707

eAppendix 1. National Pediatric Readiness Project (NPRP) Assessment With Weighted Elements

eAppendix 2. National Pediatric Readiness Project Sample (NPRP) Gap Report

eAppendix 3. National Pediatric Readiness Project (NPRP) Response Rate Map

eFigure. Emergency Department Volume Category and Pediatric Visits per Day

eTable 1. National Pediatric Readiness Project (NPRP) Response Rate Table by State

eTable 2. Comparison of Nonrespondents With Respondents of the 2021 National Pediatric Readiness Project (NPRP) Assessment

eTable 3. Comparison of Site Demographics: 2013-2021 National Pediatric Readiness Project (NPRP) Assessments

eTable 4. Comparison of Domain Scores by Pediatric Volume Categories: 2013-2021 National Pediatric Readiness Project (NPRP) Assessments

This supplemental material has been provided by the authors to give readers additional information about their work.

© 2023 Remick KE et al. *JAMA Network Open.*

eAppendix 1. National Pediatric Readiness Project (NPRP) Assessment With Weighted Elements

NATIONAL EMS FOR CHILDREN PROGRAM

PEDIATRIC READINESS ASSESSMENT AND SCORING

A Resource Document

Prepared by the National EMS for Children Data Analysis Resource Center

National
NPRP
Pediatric Readiness Project
Ensuring Emergency Care for All Children

The National Pediatric Readiness (NPRP) Assessment is based on the 2018 Policy Statement: Pediatric Readiness in the Emergency Department and was developed by NPRP collaborative partners. It is intended to be used to evaluate overall pediatric readiness in Emergency Departments. Users agree they will not adapt, alter, amend, abridge, modify, condense, make derivative works, or translate the assessment. The project is funded in part by HRSA's EMSC Data Center grant award UJ5MC30824. For more information, write to PedsReady@hsc.utah.edu.

© 2023 Remick KE et al. *JAMA Network Open*.

About the Project

The National Pediatric Readiness Project is a national multi-phase quality improvement initiative to ensure all U.S. emergency departments (EDs) have the essential guidelines and resources in place to provide effective emergency care to children. The support for this project is provided by the Emergency Medical Services (EMS) for Children Program, the American Academy of Pediatrics, the American College of Emergency Physicians, and the Emergency Nurses Association.

In 2013, the first National Pediatric Readiness Assessment was completed and results have been published and the overall data from the assessment has been studied by a number of investigators that have demonstrated that high levels of pediatric readiness result in improved outcomes for critically ill and injured children.

In May through August 2021, the second web-based assessment was implemented and over 3500 emergency departments responded representing 71% of emergency departments (EDs) in the United States (www.pedsready.org).

Upon completion of the assessment:

- Respondents received a score based on a 100-point scale representing their readiness to care for pediatric patients
- Average scores of EDs of similar pediatric volume
- Average score of all participating EDs to use as a benchmark;
- Analysis to target efforts for improvement in pediatric readiness.

The Assessment and the Score

This document includes a copy of the assessment as well as the scoring matrix that was used to generate an overall pediatric readiness score for each participating hospital. This information can be helpful for hospitals as they launch quality improvement efforts and want to track changes in their score over time.

How to Read This Document

In this document, if a question in the assessment was used as part of the pediatric readiness score, it will be followed by the number of points allotted to that question as shown in red in the example below:

> Does your ED have a physician coordinator—sometimes referred to as a pediatric emergency care coordinator (PECC) or pediatric champion—who is assigned the role of overseeing various administrative aspects of pediatric emergency care (e.g., oversees quality improvement, collaborates with nursing, ensures pediatric skills of staff, develops and periodically reviews policies)? **(Choose only one):**
>
> *Note: The physician coordinator for pediatric emergency care may have additional administrative roles in the ED.*
>
> ☐ **[9.5 points]** Our hospital has a physician coordinator that is filled by an MD or DO

This document contains similar questions from the 2013 pediatric readiness assessment, but because the guidelines have been updated in 2018 and questions modified in part, scores from the 2013 and 2021 cannot be directly compared.

© 2023 Remick KE et al. *JAMA Network Open*.

This document can be used, based on responses to all scored items, to calculate the hospital ED's pediatric readiness score. The total number of points possible is 100. A score of 100 represents the essential components needed to establish a foundation for pediatric readiness, but is in no way inclusive of all the components recommended for pediatric readiness.

Hospitals are encouraged to carefully review the nationally published guidelines "Pediatric Readiness in the Emergency Department", which served as the basis for the 2021 assessment, and to develop a comprehensive pediatric readiness program for a hospital.

Questions about the Document or the Project

If you have questions about this document, you are invited to contact the National EMS for Children Data Analysis Resource Center at 801-585-9158.

For additional information, please visit the EMS for Children Innovation and Improvement Center – National Pediatric Readiness Project at https://emscimprovement.center/domains/pediatric-readiness-project/.

© 2023 Remick KE et al. *JAMA Network Open*.

PEDIATRIC READINESS ASSESSMENT

Before we begin, please provide us with the following information, in case we need to contact you to clarify any of your responses:

1. Name: ______________________________
2. Title/Position: ______________________________
3. Phone number: ______________________________
4. Email: ______________________________
5. Name of your facility/hospital: ______________________________
6. Address of your facility/hospital: ______________________________
7. City your facility/hospital is located in: ______________________________
8. Zip code of your facility/hospital: ______________________________

From this point forward, we will use the term "hospital" to indicate a hospital or facility where your emergency department is located.

9. Does your hospital have an emergency department (ED) that is open 24/7?
   - ☐ Yes
   - ☐ No → (You do not need to complete the assessment. Thank you for your time.)

These first few questions will help us understand the infrastructure of your hospital and emergency department.

10. Which of the following best describes your hospital? (Choose one)
    - ☐ **General Hospital** (a non-specialized facility treating adults and children for all medical and trauma conditions with or without a separate pediatric ED)
    - ☐ **Children's Hospital within a General Hospital** (children's hospital located completely within a larger hospital which also sees adults)
    - ☐ **Children's Hospital** (a stand-alone, specialized facility which offers services exclusively to children and adolescents)
    - ☐ **Critical Access Hospital** (a non-specialized facility that is typically 35 miles from another hospital and maintains no more than 25 inpatient beds)
    - ☐ **Micro-Hospital** (small scale inpatient facility that typically maintains 8 to 15 beds for observation and short-stay use for low-acuity patients)
    - ☐ **Off-Site Hospital-Based or Satellite Emergency Department** (a facility providing emergency department services, basic imaging, and laboratory services)

© 2023 Remick KE et al. *JAMA Network Open*.

☐ **Independently-Owned Freestanding Emergency Department** (a stand-alone facility providing emergency department services, basic imaging, and laboratory services)

☐ **Other**

11. You answered "other," please describe your hospital:

____

____

12. Which one of the following is the best description of your ED configuration for the care of children (children as defined by your hospital)?
(Choose one)
   a. ☐ General ED (pediatric and adult patients seen in same area)
   b. ☐ Separate pediatric ED in a hospital that treats both adults and children
   c. ☐ Pediatric ED in a Children's hospital (hospital cares ONLY for children)
   d. ☐ Other

   13. You answered "other", please describe your hospital's ED configuration for the care of children: ______________________

These next questions are about your hospital's trauma designation.

14. Is your hospital designated as a trauma center?
   ☐ Yes
   ☐ No → **Skip to Question 18**

15. Which of the following are used to verify your trauma center for designation? (Check all that apply)
   a. ☐ American College of Surgeons
   b. ☐ State or Regional Level Entity (e.g., EMS authority/governing board/bureau, Department of Health)

16. At what trauma level is your hospital currently designated for adults? (Choose one)
   a. ☐ Adult Level I
   b. ☐ Adult Level II
   c. ☐ Adult Level III
   d. ☐ Adult Level IV
   e. ☐ Adult Level V
   f. ☐ None of the above

17. At what trauma level is your hospital currently designated for children? (Choose one)
   a. ☐ Pediatric Level I
   b. ☐ Pediatric Level II
   c. ☐ None of the above

© 2023 Remick KE et al. *JAMA Network Open*.

Now, we would like to ask you some questions regarding your hospital's inpatient services.

18. Which of the following inpatient services does your hospital have on-site? (Check Yes or No for each)

a. Newborn nursery	Yes ☐	No ☐
b. Neonatal intensive care unit	Yes ☐	No ☐
c. Pediatric intensive care unit	Yes ☐	No ☐
d. Pediatric step-down unit	Yes ☐	No ☐
e. Pediatric inpatient ward	Yes ☐	No ☐
f. Adult intensive care unit (medical or surgical)	Yes ☐	No ☐
g. Adult step-down unit	Yes ☐	No ☐
h. Adult inpatient ward	Yes ☐	No ☐

Please answer the following questions according to your hospital's definition of children.

If you answered yes to adult intensive care unit (medical or surgical) on Question 18:

19. Does your hospital ever admit children to the adult intensive care unit (medical or surgical)?

☐ Yes
☐ No

If you answered yes to adult step-down unit for Question 18:

20. Does your hospital ever admit children to the adult step-down unit?

☐ Yes
☐ No

If you answered yes to adult inpatient ward for Question 18:

21. Does your hospital ever admit children to the adult inpatient ward?

☐ Yes
☐ No

Administration and Coordination for the Care of Children

Answers to the following questions will help us to better understand the resources available for the care of children in your ED.

© 2023 Remick KE et al. *JAMA Network Open*.

Physician Administration/Coordination

22. Does your ED have a physician coordinator—sometimes referred to as a pediatric emergency care coordinator (PECC) or pediatric champion—who is assigned the role of overseeing various administrative aspects of pediatric emergency care (e.g., oversees quality improvement, collaborates with nursing, ensures pediatric skills of staff, develops and periodically reviews policies)? **(Choose only one):**

    Note: The physician coordinator for pediatric emergency care may have additional administrative roles in the ED.

    ☐ **[9.5 points]** Our hospital has a physician coordinator that is filled by an MD or DO

    ☐ **[9.5 points]** Our hospital has a physician coordinator that is filled by an Advanced Practice Provider (e.g., Physician Assistant or Nurse Practitioner) with physician oversight

    ☐ **[0 points]** Our hospital does NOT HAVE a physician coordinator at this time
    → **Skip to Question 25**

23. Is dedicated non-clinical time allotted to complete the tasks associated with the physician coordinator role?

    ☐ Yes
    ☐ No

24. Which of the following statements best describes the scope of the physician coordinator role? (Choose one)

    ☐ An individual who coordinates care only for your hospital's ED
    ☐ An individual who coordinates care for your hospital's ED as well as other hospitals' EDs

Nurse Administration/Coordination

25. Does your ED have a nurse coordinator—sometimes referred to as a pediatric emergency care coordinator (PECC) or pediatric champion—who is assigned the role of overseeing various administrative aspects of pediatric emergency care (e.g., facilitates continuing education, facilitates quality improvement activities, ensures pediatric specific elements are included in orientation of staff)? **(Choose only one):**

    ☐ **[9.5 points]** Our hospital has a nurse coordinator that is filled by an RN

    ☐ **[9.5 points]** Our hospital has a nurse coordinator that is filled by a Nurse Practitioner

    ☐ **[0 points]** Our hospital does NOT HAVE a nurse coordinator at this time
    → **Skip to Question 28**

26. Is dedicated non-clinical time allotted to complete the tasks associated with the nurse coordinator role?

    ☐ Yes
    ☐ No

© 2023 Remick KE et al. *JAMA Network Open*.

27. Which of the following statements best describes the scope of the nurse coordinator role? (Choose one)

☐ An individual who coordinates care only for your hospital's ED
☐ An individual who coordinates care for your hospital's ED as well as other hospitals' EDs

The following questions refer to personnel, quality improvement, and patient safety in the ED. If you have a separate pediatric ED, then answer based on resources for that area; if you do not have a separate pediatric ED, then answer based on the overall ED resources.

Personnel – Physicians

28. Is there a physician working on-site in the ED 24/7?

☐ Yes
☐ No ⟶ **Skip to Question 30**

29. If yes, what types of training/certification are required for physicians who staff your ED 24/7 and care for children?
(Check Yes or No for each)

a. Emergency medicine board eligible/certified	Yes ☐	No ☐
b. Pediatric emergency medicine board eligible/certified	Yes ☐	No ☐
c. Pediatrics board eligible/certified	Yes ☐	No ☐
d. Family medicine board eligible/certified	Yes ☐	No ☐
e. Internal medicine board eligible/certified	Yes ☐	No ☐
f. Surgery board eligible/certified	Yes ☐	No ☐
g. Board eligible/certified physician with other training	Yes ☐	No ☐
h. Non-Board eligible/certified physician with other training	Yes ☐	No ☐

30. **[2.5 points]** Does your hospital have a policy for physician credentialing that requires pediatric-specific competencies for working in the ED (e.g., continuing education requirements, maintenance of board certification, hospital specific competency evaluations)?

☐ Yes
☐ No ⟶ **Skip to Question 34**

© 2023 Remick KE et al. *JAMA Network Open*.

If yes, then which of the following are required?

31. Continuing education requirements in pediatric emergency care

☐ Yes
☐ No

32. **[2.5 points]** Maintenance of board certification

☐ Yes
☐ No

33. Hospital-specific competency evaluations (e.g., sedation and analgesia)

☐ Yes
☐ No

Personnel – Nurses

34. **[2.5 points]** Does your hospital have a policy for nurse credentialing that requires pediatric-specific competencies for working in the ED (e.g., continuing education requirements, maintenance specialty certifications, hospital specific competency evaluations)?

☐ Yes
☐ No → **Skip to Question 38**

If yes, which of the following are required?

35. Continuing education requirements in pediatric emergency care (e.g., ENPC, PALS)

☐ Yes
☐ No

36. **[2.5 points]** Maintenance of specialty certification for nurses (e.g., CEN, CPEN)

☐ Yes
☐ No

37. Hospital-specific competency evaluations (e.g., triage, pain assessment)

☐ Yes
☐ No

Personnel – Advanced Practice Providers (Nurse Practitioners, Physician Assistants)

38. Does your hospital employ advanced practice providers (nurse practitioners and/or physician assistants) to provide care for children in the ED?

☐ Yes
☐ No → **Skip to Question 43**

© 2023 Remick KE et al. *JAMA Network Open*.

39. Does your hospital staff policy for advanced practice provider credentialing require pediatric-specific competencies for working in the ED (e.g., continuing education requirements, maintenance of national specialty certification, hospital specific competency evaluations)?

☐ Yes
☐ No ⟶ **Skip to Question 43**

If yes, which of the following are required?

40. Continuing education requirements in pediatric emergency care

☐ Yes
☐ No

41. Maintenance of national specialty certification

☐ Yes
☐ No

42. Hospital specific competency evaluations (e.g., pain assessment and management)

☐ Yes
☐ No

Quality Improvement

43. Does your ED have a Quality Improvement/Performance Improvement Plan for pediatric patients? (e.g., chart review, collection of pediatric emergency care data, development of a plan to improve pediatric emergency care)

Note: This may be a separate Quality Improvement/Performance Improvement Plan for pediatric patients or integrated into the overall ED Quality Improvement/Performance Improvement Plan.

☐ Yes
☐ No ⟶ **Skip to Question 45**

44. If yes, are each of the following components included in the Quality Improvement/Performance Improvement Plan?
(Check Yes or No for each)

a. **[1.4 points]** Patient care review process (chart review) — Yes ☐ No ☐

b. **[1.4 points]** Identification of quality indicators for children (e.g., timely administration of steroids in acute asthma exacerbation or time to antibiotics in the pediatric sepsis patient) — Yes ☐ No ☐

c. **[1.4 points]** Collection and analysis of pediatric emergency care data (e.g., admissions, transfers, death in the ED, or return visits) Yes ☐ No ☐

d. **[1.4 points]** Development of a plan for improvement in pediatric emergency care (e.g., process to ensure that variances in care are addressed through education or training and reassessed for evidence of improvement) Yes ☐ No ☐

e. **[1.4 points]** Re-evaluation of performance using outcomes-based measures (e.g., how often was pain rapidly controlled or fever properly treated?) Yes ☐ No ☐

Pediatric Patient Safety in the ED

45. **[1.5 points]** Are all children seen in the ED weighed in kilograms (without conversion from pounds)? Yes ☐ No ☐
46. **[1.5 points]** Are all children's weights recorded in the ED medical record in kilograms only? Yes ☐ No ☐
47. **[1 point]** Are temperature, heart rate, and respiratory rate recorded on all children? Yes ☐ No ☐
48. **[1 point]** Is blood pressure monitoring available for children of all ages based on severity of illness? Yes ☐ No ☐
49. **[1 point]** Is pulse oximetry monitoring available for children of all ages based on severity of illness? Yes ☐ No ☐
50. **[0.5 points]** Is end tidal CO2 monitoring available for children of all ages based on severity of illness? Yes ☐ No ☐
51. **[3 points]** Is there a process in place for notification (manual or automated) of physicians when abnormal vital signs are found? Yes ☐ No ☐
52. **[3 points]** Is a process in place for the use of pre-calculated drug dosing in all children? Yes ☐ No ☐
53. **[0.5 points]** Is a process in place that allows for 24/7 access to interpreter services in the ED? Yes ☐ No ☐
54. **[0.5 points]** Is level of consciousness (e.g., AVPU or GCS) assessed in all children? Yes ☐ No ☐
55. **[0.5 points]** Is level of pain assessed in all children? Yes ☐ No ☐

© 2023 Remick KE et al. *JAMA Network Open*.

Now, we would like to know about policies and/or procedures that your ED has to address the needs of children. These pediatric policies may be integrated into the overall ED policy manual or may be listed separately. They should also be available to staff in the ED, either in written or electronic format.

Policies and Procedures

56. **[2 points]** Does your ED have a triage policy that specifically addresses ill and injured children?

☐ Yes
☐ No

57. Does your ED have any of the following policies, procedures, or plans? (Check Yes or No for each)

a. **[1.5 points]** Pediatric patient assessment and reassessment policies, procedures, or plans — Yes ☐ No ☐

b. **[1.5 points]** Immunization assessment and management of the UNDER-IMMUNIZED child policies, procedures, or plans — Yes ☐ No ☐

c. **[1.5 points]** Child maltreatment policies, procedures, or plans — Yes ☐ No ☐

d. **[1.5 points]** Death of the child in the ED policies, procedures, or plans — Yes ☐ No ☐

e. **[1.5 points]** Reduced-dose radiation for CT and x-ray imaging based on pediatric age or weight policies, procedures, or plans — Yes ☐ No ☐

f. **[1.5 points]** Behavioral health issues policies, procedures, or plans for children of all ages — Yes ☐ No ☐

58. Does your ED have a written guideline for the transfer of children with behavioral health issues out of your facility to an appropriate facility? — Yes ☐ No ☐

59. Does your ED have social services policies, procedures, or a plan for children of all ages? — Yes ☐ No ☐

Policies for Family-Centered Care

60. Does your ED have a policy for promoting family-centered care? (e.g., family presence, family involvement in clinical decision making)

☐ Yes
☐ No ⟶ **Skip to Question 62**

© 2023 Remick KE et al. *JAMA Network Open*.

61. If yes, does your ED's family-centered care policy include any of the following? (Check Yes or No for each)

a. **[0.4 points]** Involving families and caregivers in patient care decision-making — Yes ☐ No ☐

b. **[0.4 points]** Involving families and caregivers in medication safety processes — Yes ☐ No ☐

c. **[0.4 points]** Family and guardian presence during all aspects of emergency care, including resuscitation — Yes ☐ No ☐

d. **[0.4 points]** Education of the patient, family, and caregivers on treatment plan and disposition — Yes ☐ No ☐

e. **[0.4 points]** Bereavement counseling — Yes ☐ No ☐

Policies for Disaster Planning

62. Does your hospital disaster plan address issues specific to the care of children (e.g., pediatric surge capacity, patient tracking and reunification, pediatric decontamination)?

☐ Yes
☐ No → **Skip to Question 68**

63. If yes, does your hospital disaster plan include each of the following? (Check Yes or No for each)

a. **[0.29 points]** Availability of medications, vaccines (e.g., tetanus and influenza), equipment, supplies, and appropriately trained providers for children in disasters — Yes ☐ No ☐

b. **[0.29 points]** Decontamination, isolation, and quarantine of families and children of all ages — Yes ☐ No ☐

c. **[0.29 points]** Minimization of parent-child separation and methods for reuniting separated children with their families — Yes ☐ No ☐

d. **[0.29 points]** All disaster drills include pediatric patients — Yes ☐ No ☐

64. **[0.28 points]** Pediatric surge capacity for both injured and non-injured children — Yes ☐ No ☐

65. **[0.28 points]** Access to behavioral health resources for children in the event of a disaster — Yes ☐ No ☐

66. Access to social services for children in the event of a disaster — Yes ☐ No ☐

67. **[0.28 points]** The care of children with special health care needs, including children with developmental disabilities — Yes ☐ No ☐

Next, we would like to know about your hospital's interfacility transfer guidelines.

68. **[2 points]** Does your hospital have written interfacility guidelines that outline procedural and administrative policies with other hospitals for the transfer of patients of all ages including children in need of care not available at your hospital?

Note: Compliance with EMTALA does not constitute having interfacility transfer guidelines. The guidelines may be a separate document or part of an interfacility transfer agreement document.

☐ Yes
☐ No ⟶ **Skip to Question 70**

69. You answered that your hospital has written interfacility transfer guidelines. Please indicate whether the guidelines include the information specifically for the transfer of patients for each item below. (Check Yes or No for each)

a.	Defined process for initiation of transfer, including the roles and responsibilities of the referring facility and referral center (including responsibilities for requesting transfer and communication)	Yes ☐	No ☐
b.	Process for selecting the appropriate care facility	Yes ☐	No ☐
c.	Process for selecting the appropriately staffed transport service to match the patient's acuity level (e.g., level of care required by patient or equipment needed in transport)	Yes ☐	No ☐
d.	Process for patient transfer (including obtaining informed consent)	Yes ☐	No ☐
e.	Plan for transfer of copy of patient medical record	Yes ☐	No ☐
f.	Plan for transfer of a copy of the signed transport consent	Yes ☐	No ☐
g.	Plan for transfer of personal belongings of the patient	Yes ☐	No ☐
h.	Plan for provision of directions and referral institution information to family	Yes ☐	No ☐

Now, we would like to know about your hospital's interfacility transfer agreements.

70. Does your hospital have written interfacility agreement(s) with other hospitals for the transfer of patients of all ages including children in need of care not available at your hospital?

    Note: Compliance with EMTALA does not constitute having interfacility transfer agreements. Agreements may be a separate document or part of an interfacility transfer guidelines document.

    ☐ Yes
    ☐ No

We would like to know about the equipment and supplies for children in your ED and how they are stored and resupplied.

Equipment and Supplies Management

71. **[3 points]** Are all ED staff trained on the location of all pediatric equipment and medications?

    ☐ Yes
    ☐ No

72. **[3 points]** Is there a daily method used to verify the proper location and stocking of pediatric equipment and supplies?

    ☐ Yes
    ☐ No

73. **[3 points]** Is there a standardized chart or tool to estimate weight if resuscitation precludes the use of a weight scale (e.g., length-based tape)?

    ☐ Yes
    ☐ No

Monitoring Equipment

74. Are each of the following monitoring equipment items available for immediate use in the ED?
    (Check Yes or No for each)

    a. **[0.5 points]** Neonatal blood pressure cuff Yes ☐ No ☐
    b. **[0.5 points]** Infant blood pressure cuff Yes ☐ No ☐
    c. **[0.5 points]** Child blood pressure cuff Yes ☐ No ☐
    d. **[0.5 points]** Defibrillator with pediatric and adult capabilities including pads and/or paddles Yes ☐ No ☐
    e. **[0.5 points]** Pulse oximeter with pediatric and adult probes Yes ☐ No ☐

f. **[0.5 points]** Continuous end-tidal CO_2 monitoring device — Yes ☐ No ☐

Resuscitation Equipment

75. Are each of the following fluid resuscitation equipment items available for immediate use in the ED?
(Check Yes or No for each)

a. **[0.5 points]** 22 gauge catheter-over-the-needle — Yes ☐ No ☐

b. **[0.5 points]** 24 gauge catheter-over-the-needle — Yes ☐ No ☐

c. **[0.5 points]** Pediatric intra-osseus needles — Yes ☐ No ☐

d. **[0.5 points]** IV administration sets with calibrated chambers or an infusion pump with the ability to regulate rate and volume of infusate (e.g., buretrol) — Yes ☐ No ☐

Airway Equipment

76. Are each of the following respiratory/airway management equipment items available for immediate use in the ED?
(Check Yes or No for each)

a. **[0.575 points]** Endotracheal tubes: cuffed or uncuffed 2.5 mm — Yes ☐ No ☐

b. **[0.575 points]** Endotracheal tubes: cuffed or uncuffed 3.0 mm — Yes ☐ No ☐

c. **[0.575 points]** Endotracheal tubes: cuffed or uncuffed 3.5 mm — Yes ☐ No ☐

d. **[0.575 points]** Endotracheal tubes: cuffed or uncuffed 4.0 mm — Yes ☐ No ☐

e. **[0.575 points]** Endotracheal tubes: cuffed or uncuffed 4.5 mm — Yes ☐ No ☐

f. **[0.575 points]** Endotracheal tubes: cuffed or uncuffed 5.0 mm — Yes ☐ No ☐

g. **[0.575 points]** Endotracheal tubes: cuffed or uncuffed 5.5 mm — Yes ☐ No ☐

h. **[0.575 points]** Endotracheal tubes: cuffed 6.0 mm — Yes ☐ No ☐

77. Are each of the following respiratory/airway management equipment items available for immediate use in the ED?
(Check Yes or No for each)

a. **[0.576 points]** Laryngoscope blades: straight, size 0 — Yes ☐ No ☐

© 2023 Remick KE et al. *JAMA Network Open*.

b. **[0.576 points]** Laryngoscope blades: straight, size 1 Yes ☐ No ☐

c. **[0.576 points]** Laryngoscope blades: straight, size 2 Yes ☐ No ☐

d. **[0.576 points]** Laryngoscope blades: curved, size 2 Yes ☐ No ☐

e. **[0.576 points]** Pediatric-sized Magill forceps Yes ☐ No ☐

f. **[0.576 points]** Nasopharyngeal airways: infant-sized Yes ☐ No ☐

g. **[0.576 points]** Nasopharyngeal airways: child-sized Yes ☐ No ☐

h. **[0.576 points]** Oropharyngeal airways: size 0 Yes ☐ No ☐

i. **[0.576 points]** Oropharyngeal airways: size 1 (60mm) Yes ☐ No ☐

j. **[0.576 points]** Oropharyngeal airways: size 2 (70mm) Yes ☐ No ☐

k. **[0.576 points]** Oropharyngeal airways: size 3 (80mm) Yes ☐ No ☐

l. **[0.576 points]** Stylets for pediatric/infant-sized endotracheal tubes Yes ☐ No ☐

78. Are each of the following respiratory/airway management equipment items available for immediate use in the ED?
(Check Yes or No for each)

a. **[0.576 points]** Bag-mask device, self-inflating (infant/child) Yes ☐ No ☐

b. **[0.576 points]** Masks (neonatal size) to fit bag-mask device Yes ☐ No ☐

c. **[0.576 points]** Masks (infant size) to fit bag-mask device Yes ☐ No ☐

d. **[0.576 points]** Masks (child size) to fit bag-mask device Yes ☐ No ☐

e. **[0.576 points]** Simple oxygen face masks: standard infant Yes ☐ No ☐

f. **[0.576 points]** Clear oxygen masks: standard child Yes ☐ No ☐

g. **[0.576 points]** Non-rebreather masks: infant-sized Yes ☐ No ☐

h. **[0.576 points]** Non-rebreather masks: child-sized Yes ☐ No ☐

i. **[0.576 points]** Nasal cannulas: infant Yes ☐ No ☐

j. **[0.576 points]** Nasal cannulas: child Yes ☐ No ☐

k. **[0.576 points]** Suction catheters: at least one in range 6-8F — Yes ☐ No ☐

l. **[0.576 points]** Suction catheters: at least one in range 10-12F — Yes ☐ No ☐

m. **[0.576 points]** Supplies/kit for pediatric patients with difficult airways (e.g., supraglottic airways, needle cricothyrotomy supplies, surgical cricothyrotomy kit, and/or video laryngoscopy) — Yes ☐ No ☐

Please provide actual data or estimations of ED patient volume for the following:

79. Estimate the total number of patients (adult and pediatric) seen in your ED in the last year. (Numeric data only, e.g., 5000, not "five thousand")

    Number of Total Patients: ________________________

80. Estimate the number of pediatric patients (as defined by your hospital) seen in your ED in the last year.
    (Choose one)

    - ☐ Low: <1,800 pediatric patients (average of 5 or fewer a day)
    - ☐ Medium: 1,800 – 4,999 pediatric patients (average of 6-13 a day)
    - ☐ Medium to High: 5,000 – 9,999 pediatric patients (average of 14-26 a day)
    - ☐ High: >=10,000 pediatric patients (average of 27 or more a day)

81. If you know the actual number or a more precise estimate of pediatric patients seen in your ED in the last year, please record the number below. (Numeric data only, e.g., 500, not "five hundred")

    Number of Pediatric Patients: __________________

Answers to the following questions will help us target efforts of the National Pediatric Readiness Project Coalition.

Helpful Resources

82. Please choose the resources which you feel are needed to support the role of pediatric emergency care coordinators. (Check all that apply) (not required)

    - ☐ Job descriptions for pediatric emergency care coordinators (PECC)
    - ☐ Talking points for administration to support all pediatric readiness efforts, including the role of the PECC, in your facility
    - ☐ Specialized training for PECCs
    - ☐ Other

© 2023 Remick KE et al. *JAMA Network Open*.

83. You answered “other”, please describe: ________________________________

84. Please choose the resources which you feel are needed to improve pediatric emergency care. (Check all that apply) (not required)

- ☐ Access to evidence-based clinical pathways for children
- ☐ Template for a pediatric Quality Improvement Plan
- ☐ Template for all hazards disaster plans for children
- ☐ Template for pediatric surge planning
- ☐ Model policies and procedures for care of children
- ☐ Other

85. You answered “other”, please describe: ________________________________

86. Please choose the resources which you feel are needed to improve staff comfort/preparedness to care for children in the ED. (Check all that apply) (not required)

- ☐ Access to education for all staff caring for children (e.g., educational webinars/learning modules)
- ☐ Incentives for staff who improve pediatric emergency care for children in your ED
- ☐ Access to simulation/mock codes to care for children
- ☐ Access to team training with all health care providers impacting pediatric emergency care
- ☐ Other

87. You answered “other”, please describe: ________________________________

88. Please choose the resources which you feel are needed to improve ED infrastructure. (Check all that apply) (not required)

- ☐ Optimization of electronic medical records to facilitate patient safety (e.g., calculation of dosing to reduce error, vital signs or symptom-based alerts)
- ☐ Optimization of equipment to facilitate patient safety (e.g., weight scales)
- ☐ Other

89. You answered “other”, please describe: ________________________________

90. Please choose the EXTERNAL resources which you feel are needed to improve the pediatric readiness of your ED. (Check all that apply) (not required)

- ☐ Cost-calculator for items in the 2018 Pediatric Readiness Guidelines
- ☐ Access to pediatric expertise – regionally through tele-medicine
- ☐ Development of a regional, state or national network for pediatric emergency care coordinators to share resources and best practices
- ☐ Model plan for regional or state recognition of facilities for pediatric readiness

© 2023 Remick KE et al. *JAMA Network Open*.

☐ Opportunities to participate in quality improvement collaborative(s)

☐ Other

91. You answered “other”, please describe: ________________________________

92. If you have any comments regarding pediatric readiness, please note them here:

____

____

Thank you for completing this important assessment!

© 2023 Remick KE et al. *JAMA Network Open*.

eAppendix 2. National Pediatric Readiness Project Sample (NPRP) Gap Report

Pediatric Readiness Assessment Gap Report

Report Generated Date: 5/6/2023 12:11:25 PM

National PRP Pediatric Readiness Project
Ensuring Emergency Care for All Children

Hospital Name: [redacted]

Hospital Volume: Low: <1,800 pediatric patients (average of 5 or fewer a day)

Current Assessment Date: 05/10/2021

Respondent Name: [redacted]

Respondent Contact Info: [redacted]

Previous Assessment Date: 04/16/2013

Respondent Name: [redacted]

Respondent Contact Info: [redacted]

We encourage you to **export this Gap Report to a pdf** as you **will not have access** to the report after exiting this screen (see the button above). If you have any questions about the report, please contact our support team via email at pedsready@hsc.utah.edu.

Below, in the box on the left, is the Pediatric Readiness Score for your Emergency Department (ED). The other boxes allow you to compare your score to other EDs in the nation. Your score represents the essential components to establish a foundation for pediatric readiness. The score does not include all of the components recommended for pediatric readiness. Please review the Guidelines for Care of Children in the Emergency Department to develop a comprehensive pediatric readiness program for your Emergency Department. Other important resources include a Pediatric Readiness Resource Toolkit and the Health Resources and Services Administration (HRSA) Critical Crossroads Toolkit: HRSA Critical Crossroads Toolkit Pediatric Mental Health Care in the Emergency Department.

YOUR PEDIATRIC READINESS SCORE COMPARED TO THE NATION:

86	66<br>n=1605	71<br>n=3178
YOUR CURRENT SCORE OUT OF 100 (05/10/2021)	NATIONAL AVERAGE SCORE OF EDS WITH SIMILAR PEDIATRIC VOLUME	NATIONAL AVERAGE SCORE OF ALL PARTICIPATING HOSPITALS

© 2023 Remick KE et al. *JAMA Network Open*.

ANALYSIS OF QUESTIONS IN THE ASSESSMENT BY SECTION

The following analysis is grouped by the **six** main sections of the assessment. Each section has an overall section score based on the current assessment. Under the section headings are the lists for each of the scored questions in that section along with a comparison between the previous (if applicable) and current assessment periods. **If this is the first time you have participated in the assessment, you will see an "N/A" in the previous assessment column.**

If a question has been added since the original 2013-14 national assessment **you will see an "N/A" in the previous assessment column**, indicating that question was not available during the previous assessment.

The **"Readiness"** column is included to provide a quick view of whether or not your ED currently meets each metric. The column provides a visual to help prioritize areas for improvement.
**The sum of the sectional scores below may vary slightly from your actual overall readiness score above due to rounding.*

LEGEND:

✓ **Readiness Met** ⊖ **Do Not Have Item**

Guidelines for Administration and Coordination of the ED for the Care of Children

YOUR SCORE: 19.0 out of 19

	PREVIOUS: 04/16/2013	CURRENT: 05/10/2021	Points Possible	Readiness
**Physician Coordinator**	0.0	9.5	9.5	✓
**Nurse Coordinator**	9.5	9.5	9.5	✓

Physicians, Nurses, and Other Health Care Providers Who Staff the ED

YOUR SCORE: 7.5 out of 10

	PREVIOUS: 04/16/2013	CURRENT: 05/10/2021	Points Possible	Readiness
**Physician Competency Evaluations**	2.5	2.5	2.5	✓
**Physician Maintenance of Board Certification**	N/A	2.5	2.5	✓

© 2023 Remick KE et al. *JAMA Network Open*.

Nurse Competency Evaluations	0.0	2.5	2.5	✓
Nurse Maintenance of Specialty Certification	N/A	0.0	2.5	⛔

- You indicated that maintenance of specialty certification for nurses IS NOT required of nurses staffing the ED.

  IMPORTANCE: Credentialing for nursing staff who see pediatric patients is important to ensure critical staff stays up-to-date on current treatment guidelines and protocols, and for maintenance of psychomotor skills necessary to care for this population of patients.

Guidelines QI/PI in the ED

YOUR SCORE:
7.0 out of 7

	PREVIOUS: 04/16/2013	CURRENT: 05/10/2021	Points Possible	Readiness
Patient care-review process (chart review)	1.4	1.4	1.4	✓
Identification of quality indicators for children	1.4	1.4	1.4	✓
Collection and analysis of pediatric emergency care data	1.4	1.4	1.4	✓
Development of a plan for improvement in pediatric emergency care	1.4	1.4	1.4	✓
Re-evaluation of performance using outcomes-based measures	1.4	1.4	1.4	✓

Guidelines for Improving Pediatric Patient Safety in the ED

YOUR SCORE:
12.5 out of 14

	PREVIOUS: 04/16/2013	CURRENT: 05/10/2021	Points Possible	Readiness
Children seen in the ED weighed in kg (without conversion from pounds)	1.5	1.5	1.5	✓
Children's weights recorded in the ED medical record in kg only	1.5	0.0	1.5	⛔
Temperature, heart rate, and respiratory rate recorded	1.0	1.0	1.0	✓

© 2023 Remick KE et al. *JAMA Network Open*.

Blood pressure monitoring available based on severity of illness	1.0	1.0	1.0	✓
Pulse oximetry monitoring available based on severity of illness	1.0	1.0	1.0	✓
End-tidal CO2 monitoring available based on severity of illness	N/A	0.5	0.5	✓
Process in place for notification (manual or automated) of physicians when abnormal vital signs are found	3.0	3.0	3.0	✓
Process in place for the use of pre-calculated drug dosing in all children	0.0	3.0	3.0	✓
Process in place that allows for 24/7 access to interpreter services in the ED	0.5	0.5	0.5	✓
Level of consciousness (e.g., AVPU or GCS) assessed in all children	N/A	0.5	0.5	✓
Level of pain assessed in all children	N/A	0.5	0.5	✓

- You DID NOT indicate that all children's weights are recorded in the ED medical record in kg only.

  IMPORTANCE: One of the most important safety initiatives for children as determined by a number of national medical professional organizations is weighing and recording weight in kg. Measuring in kg ensures that dosing is based on accurate weight and not subject to calculation error, which can lead to serious adverse events.

Guidelines for Policies, Procedures, and Protocols for the ED

YOUR SCORE:
6.7 out of 17

	PREVIOUS: 04/16/2013	CURRENT: 05/10/2021	Points Possible	Readiness
Triage policy that specifically addresses ill and injured children	0.00	0.00	2.00	−
Policy for pediatric patient assessment and reassessment	1.50	1.50	1.50	✓
Policy for immunization assessment and management of the under-immunized child	1.50	0.00	1.50	−
Policy for child maltreatment	1.50	1.50	1.50	✓

© 2023 Remick KE et al. *JAMA Network Open*.

Policy for death of the child in the ED	0.00	0.00	1.50	−
Policy for reduced-dose radiation for CT and x-ray imaging based on pediatric age or weight	0.00	0.00	1.50	−
Policy for behavioral health issues for children of all ages	N/A	0.00	1.50	−
Involving families and caregivers in patient care decision-making	N/A	0.00	0.40	−
Involving families and caregivers in medication safety processes	N/A	0.00	0.40	−
Family and guardian presence during all aspects of emergency care, including resuscitation	N/A	0.00	0.40	−
Education of the patient, family, and caregivers on treatment plan and disposition	N/A	0.00	0.40	−
Bereavement counseling	N/A	0.00	0.40	−
Disaster plan includes availability of medications, vaccines, equipment, supplies, and appropriately trained providers for children	N/A	0.29	0.29	✓
Disaster plan includes decontamination, isolation, and quarantine of families and children	N/A	0.29	0.29	✓
Disaster plan includes minimization of parent-child separation and methods for reuniting separated children with their families	N/A	0.29	0.29	✓
All disaster drills include pediatric patients	N/A	0.29	0.29	✓
Disaster plan includes pediatric surge capacity for both injured and non-injured children	N/A	0.28	0.28	✓
Disaster plan includes access to behavioral health resources for children	N/A	0.28	0.28	✓
Disaster plan includes care of children with special health care needs	N/A	0.00	0.28	−
Written interfacility transfer guidelines	2.00	2.00	2.00	✓

© 2023 Remick KE et al. *JAMA Network Open*.

- You indicated that your ED DOES NOT have a triage policy that specifically addresses ill and injured children.

  IMPORTANCE: Triage schemes (e.g., Emergency Severity Index algorithm or ESI) which address the special needs of children is vital to appropriate prioritization of patients to be seen.

- You indicated that your ED DOES NOT have policies or procedures for immunization assessment and management of the under-immunized child.

  IMPORTANCE: Immunizations have changed the epidemiology of pediatric infectious disease in the United States. Assessment of risk from lack of immunization is an important part of the assessment process for children and helps to identify children at increased risk for certain diseases.

- You indicated that your ED DOES NOT have policies or procedures for death of the child in the ED.

  IMPORTANCE: A child's death has a profound effect on families. It is essential to have a policy that addresses the needs of the family in order to minimize confusion and assist with the grieving and healing processes.

- You indicated that your ED DOES NOT have policies or procedures for reduced-dose radiation for CT and x-ray imaging based on pediatric age or weight.

  IMPORTANCE: Adult imaging protocols transmit excessive radiation to children. Weight-based or age-based protocols for adjusting the radiation exposure for children can ensure adequate imaging while minimizing radiation exposure and subsequent risk of cancer.

- You indicated that your ED DOES NOT have policies or procedures for behavioral health issues for children of all ages.

  IMPORTANCE: Behavioral and psychological health processes for children should be clearly outlined so as to address them in a timely and appropriate manner within the context of the incident. Plans should also include a way to obtain further mental health resources for children in crisis when those available locally are not adequate. Behavioral health may not be the primary reason for an emergency visit, but should be assessed on all children, as developmentally appropriate, to include risk assessment for suicidality/homicidality, abuse and/or neglect.

- You indicated that your ED's family-centered policy DOES NOT involve families and caregivers in patient care decision-making.

  IMPORTANCE: It is necessary to have a shared decision-making model with caregivers to achieve

an agreed upon outcome. This helps to ensure families feel their concerns are addressed and helps facilitate the transition of care back to the family. Families, especially those with children with special health care needs, often are more knowledgeable about what works well for their child than a provider who is seeing them for the first time.

- You indicated that your ED's family-centered policy DOES NOT involve families and caregivers in medication safety processes.

  IMPORTANCE: Family-centered care is not only critical during treatment decisions and resuscitations, but should be integrated throughout the healthcare visit. Children are at high risk of experiencing medication errors compared to adults. Integrating families into the medication administration process can help to ensure the correct medication and dose are given and that there are fewer medication errors.

- You indicated that your ED's family-centered policy DOES NOT include family and guardian presence during all aspects of emergency care, including resuscitation.

  IMPORTANCE: Caregivers are often the most knowledgeable of a child's past medical/surgical history, and family presence helps to ensure treatment decisions are aligned with family values. It has also been shown that family being present during care alleviates anxiety for both patient and caregiver, and that family presence during a resuscitation that ends in death of the child is an important part of the healing process for families.

- You indicated that your ED's family-centered policy DOES NOT include education of the patient, family, and caregivers on treatment plan and disposition.

  IMPORTANCE: Inclusion of patient and caregivers in medical decision-making allows for transparency of the treatment plan, decreases medical errors, and gives opportunity for clarification and questions.

- You indicated that your ED's family-centered policy DOES NOT include bereavement counseling.

  IMPORTANCE: When there is the loss of a loved one, specific personnel should be identified and trained in how to deal with the unique challenges of discussing trauma and loss with children and families in a developmentally appropriate manner.

- You indicated that your hospital disaster plan DOES NOT include the care of children with special health care needs, including children with developmental disabilities.

  IMPORTANCE: There is a high medical utilization rate of children with special needs. Although difficult to plan for, a systematic approach should be used when encountering these patients and addressing their needs, including ensuring staff training, the availability of special equipment, and

© 2023 Remick KE et al. *JAMA Network Open*.

the importance of family-centered care. It may be useful to engage pediatricians and other specialists in the community when developing such a policy.

Guidelines for Equipment, Supplies, and Medications for the Care of Pediatric Patients in the ED

YOUR SCORE:
33.0 out of 33

	PREVIOUS: 04/16/2013	CURRENT: 05/10/2021	Points Possible	Readiness
All staff trained on the location of all pediatric equipment and medications	3.000	3.000	3.000	✓
Daily method used to verify the proper location and function of pediatric equipment and supplies	0.000	3.000	3.000	✓
Standardized chart or tool to estimate weight if resuscitation precludes the use of a weight scale (e.g., length-based tape)	3.000	3.000	3.000	✓
Neonatal blood pressure cuff	0.500	0.500	0.500	✓
Infant blood pressure cuff	0.500	0.500	0.500	✓
Child blood pressure cuff	0.500	0.500	0.500	✓
Defibrillator with pediatric and adult capabilities including pads and/or paddles	0.500	0.500	0.500	✓
Pulse oximeter with pediatric and adult probes	0.500	0.500	0.500	✓
Continuous end-tidal CO2 monitoring device	0.500	0.500	0.500	✓
22 gauge catheter-over-the-needle	0.500	0.500	0.500	✓
24 gauge catheter-over-the-needle	0.500	0.500	0.500	✓
Pediatric intra-osseus needles	0.500	0.500	0.500	✓
IV administration sets with calibrated chambers or an infusion pump	0.500	0.500	0.500	✓

© 2023 Remick KE et al. *JAMA Network Open*.

Endotracheal tubes: cuffed or uncuffed 2.5 mm	0.575	0.575	0.575	✓
Endotracheal tubes: cuffed or uncuffed 3.0 mm	0.575	0.575	0.575	✓
Endotracheal tubes: cuffed or uncuffed 3.5 mm	0.575	0.575	0.575	✓
Endotracheal tubes: cuffed or uncuffed 4.0 mm	0.575	0.575	0.575	✓
Endotracheal tubes: cuffed or uncuffed 4.5 mm	0.575	0.575	0.575	✓
Endotracheal tubes: cuffed or uncuffed 5.0 mm	0.575	0.575	0.575	✓
Endotracheal tubes: cuffed or uncuffed 5.5 mm	0.575	0.575	0.575	✓
Endotracheal tubes: cuffed 6.0 mm	0.575	0.575	0.575	✓
Laryngoscope blades: straight, size 0	0.576	0.576	0.576	✓
Laryngoscope blades: straight, size 1	0.576	0.576	0.576	✓
Laryngoscope blades: straight, size 2	0.576	0.576	0.576	✓
Laryngoscope blades: curved, size 2	0.576	0.576	0.576	✓
Pediatric-sized Magill forceps	0.576	0.576	0.576	✓
Nasopharyngeal airways: infant-size	0.576	0.576	0.576	✓
Nasopharyngeal airways: child-size	0.576	0.576	0.576	✓
Oropharyngeal airways: size 0 (50mm)	0.576	0.576	0.576	✓
Oropharyngeal airways: size 1 (60mm)	0.576	0.576	0.576	✓
Oropharyngeal airways: size 2 (70mm)	0.576	0.576	0.576	✓
Oropharyngeal airways: size 3 (80mm)	0.576	0.576	0.576	✓

© 2023 Remick KE et al. *JAMA Network Open*.

**Stylets for pediatric/infant-sized endotracheal tubes**	**0.576**	**0.576**	**0.576**	✓
**Bag-mask device, self-inflating (infant/child)**	**0.576**	**0.576**	**0.576**	✓
**Masks (neonatal size) to fit bag-mask device**	**0.576**	**0.576**	**0.576**	✓
**Masks (infant size) to fit bag-mask device**	**0.576**	**0.576**	**0.576**	✓
**Masks (child size) to fit bag-mask device**	**0.576**	**0.576**	**0.576**	✓
**Simple oxygen masks: standard infant**	**0.576**	**0.576**	**0.576**	✓
**Clear oxygen masks: standard child**	**0.576**	**0.576**	**0.576**	✓
**Non-rebreather masks: infant-sized**	**0.576**	**0.576**	**0.576**	✓
**Non-rebreather masks: child-size**	**0.576**	**0.576**	**0.576**	✓
**Nasal cannulas: infant**	**0.576**	**0.576**	**0.576**	✓
**Nasal cannulas: child**	**0.576**	**0.576**	**0.576**	✓
**Suction catheters: at least one in range 6-8F**	**0.576**	**0.576**	**0.576**	✓
**Suction catheters: at least one in range 10-12F**	**0.576**	**0.576**	**0.576**	✓
**Supplies/kit for pediatric patients with difficult airways**	**0.576**	**0.576**	**0.576**	✓

© 2023 Remick KE et al. *JAMA Network Open*.

eAppendix 3. National Pediatric Readiness Project (NPRP) Response Rate Map

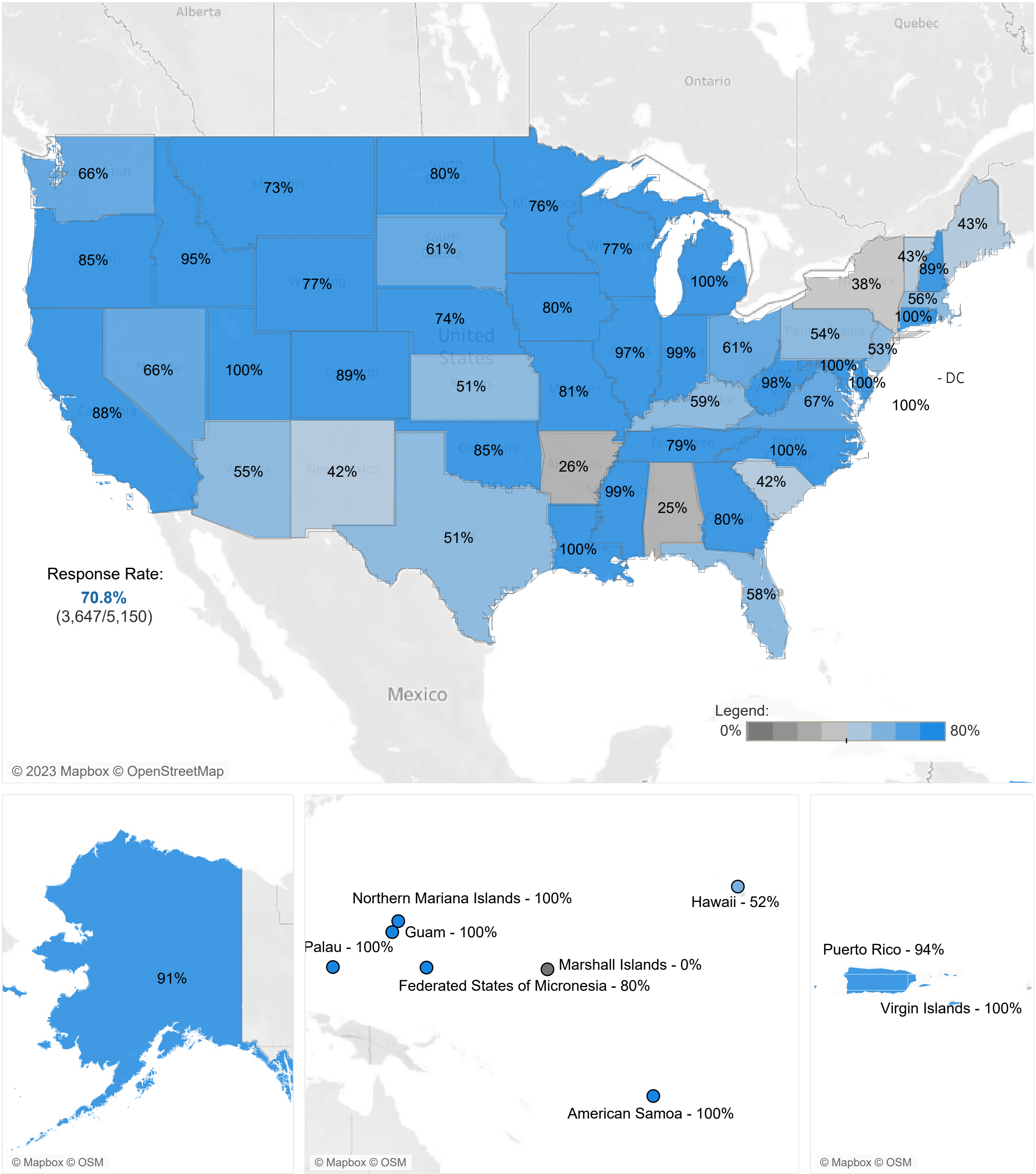

© 2023 Remick KE et al. *JAMA Network Open*.

eFigure. Emergency Department Volume Category and Pediatric Visits per Day

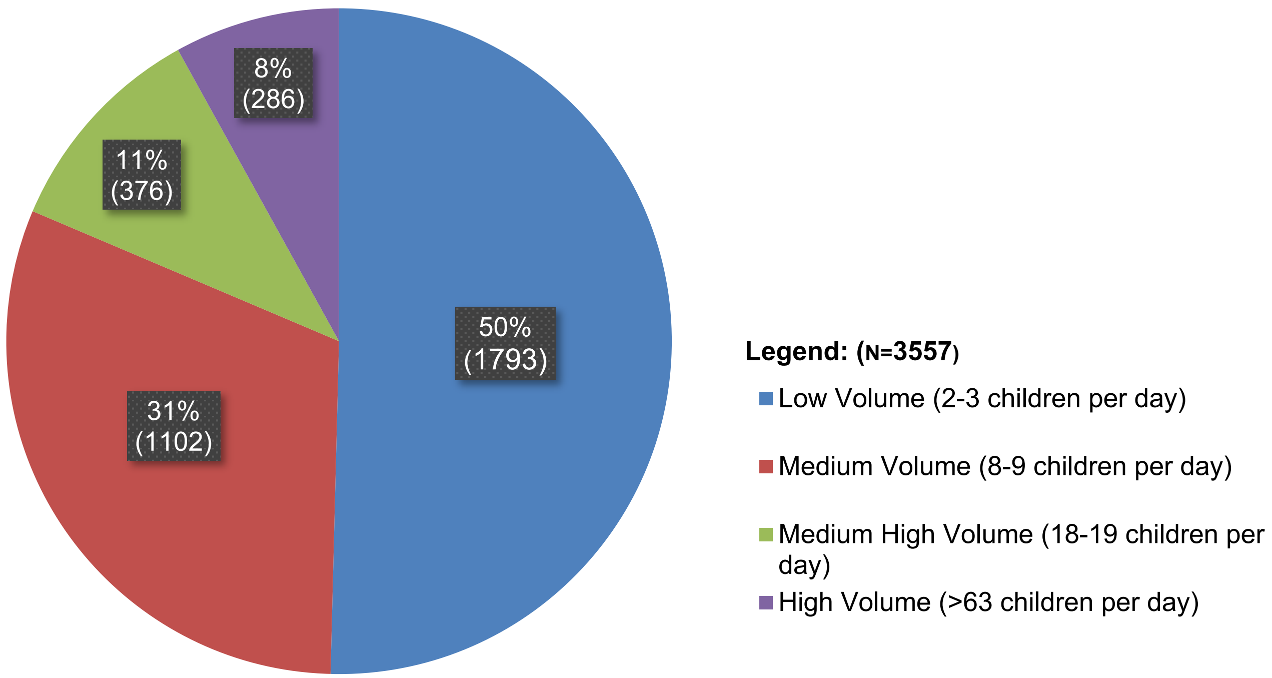

© 2023 Remick KE et al. *JAMA Network Open*.

eTable 1. National Pediatric Readiness Project (NPRP) Response Rate Table by State

State/Territory	Numerator	Denominator	Response Rate
Alabama	24	95	25.3%
Alaska	21	23	91.3%
American Samoa	1	1	100.0%
Arizona	47	86	54.7%
Arkansas	19	73	26.0%
California	293	333	88.0%
Colorado	78	88	88.6%
Connecticut	36	36	100.0%
Delaware	10	10	100.0%
District of Columbia	7	7	100.0%
Federated States of Micronesia	4	5	80.0%
Florida	170	295	57.6%
Georgia	109	136	80.1%
Guam	2	2	100.0%
Hawaii	13	25	52.0%
Idaho	38	40	95.0%
Illinois	179	184	97.3%
Indiana	132	134	98.5%
Iowa	94	118	79.7%
Kansas	71	140	50.7%
Kentucky	59	100	59.0%
Louisiana	109	109	100.0%
Maine	15	35	42.9%
Marshall Islands	0	2	0.0%
Maryland	49	49	100.0%
Massachusetts	37	66	56.1%
Michigan	138	138	100.0%
Minnesota	99	130	76.2%
Mississippi	82	83	98.8%
Missouri	91	113	80.5%
Montana	45	62	72.6%
Nebraska	62	84	73.8%
Nevada	23	35	65.7%
New Hampshire	25	28	89.3%
New Jersey	37	70	52.9%
New Mexico	18	43	41.9%
New York	73	194	37.6%
North Carolina	120	120	100.0%
North Dakota	35	44	79.5%
Northern Mariana Islands	1	1	100.0%
Ohio	113	185	61.1%

© 2023 Remick KE et al. *JAMA Network Open*.

State/Territory	Numerator	Denominator	Response Rate
Oklahoma	99	116	85.3%
Oregon	50	59	84.7%
Palau	1	1	100.0%
Pennsylvania	91	169	53.8%
Puerto Rico	48	51	94.1%
Rhode Island	9	9	100.0%
South Carolina	30	71	42.3%
South Dakota	33	54	61.1%
Tennessee	91	115	79.1%
Texas	267	525	50.9%
Utah	49	49	100.0%
Vermont	6	14	42.9%
Virgin Islands	3	3	100%
Virginia	66	98	67.3%
Washington	59	90	65.6%
West Virginia	45	46	97.8%
Wisconsin	101	132	76.5%
Wyoming	20	26	76.9%
**Grand Total**	**3,647**	**5,150**	**70.8%**

© 2023 Remick KE et al. *JAMA Network Open*.

eTable 2: Comparison of Nonrespondents With Respondents of the 2021 National Pediatric Readiness Project (NPRP) Assessment[a]

Responded to Assessment	Urban	Urban %	Suburban	Suburban %	Rural	Rural %	Remote	Remote %	Islands	Islands %	Total
Non-Respondents	965	64.2%	157	10.4%	252	16.8%	126	8.4%	3	0.2%	1503
Respondents	2299	63.0%	307	8.4%	686	18.8%	343	9.4%	12	0.3%	3647
**Total:**	**3264**		**464**		**938**		**469**		**15**		**5150**

[a]A chi-square test from the 2021 assessment suggested a non-significant relationship between geo-location and response, meaning that agencies were not more or less likely to respond to the NPRP survey based on their geo-location ($P = 0.05$)

© 2023 Remick KE et al. *JAMA Network Open*.

eTable 3. Comparison of Site Demographics: 2013-2021 NPRP Assessments

	2013 Assessment	2021 Assessment
**Urbanicity**		
Urban	2438 (58.8%)	2239 (62.9%)
Suburban	382 (9.2%)	300 (8.4%)
Rural	833 (20.1%)	674 (18.9%)
Remote	493 (11.9%)	344 (9.7%)
**Emergency Department Configuration[a]**		
General ED	3526 (85.0%)	3217 (90.4%)
Separate pediatric ED in a hospital that treats both adults and children	235 (5.7%)	222 (6.2%)
Pediatric ED in a Children's Hospital (hospital cares only for children	90 (2.2%)	94 (2.6%)
Other	295 (7.1%)	22 (0.6%)
**In-Patient Services[b]**		
Newborn nursery	1931 (57.3%)	2001 (56.3%)
Neonatal intensive care unit	951 (28.2%)	991 (27.9%)
Pediatric intensive care unit	420 (12.5%)	344 (9.7%)
Pediatric inpatient ward/unit	1798 (53.4%)	1094 (30.8%)
Adult intensive care unit (admits children)	1224 (36.3%)	632 (26.7%)
Adult inpatient ward/unit (admits children)	2317 (68.8%)	1545 (48.3%)
**Physician certification/training[c]**	**Represented in ED**	**Available in ED 24/7**
Emergency medicine board eligible/certified	3426 (82.6%)	2704 (84.1%)
Pediatric emergency medicine board eligible/certified	604 (14.6%)	454 (14.1%)
Pediatrics board eligible/certified	793 (19.1%)	406 (12.6%)
Family medicine board eligible/certified	2561 (61.8%)	948 (29.5%)
Physician with other training board eligible/certified or non-board eligible/certified	1118 (27.0%)	910 (28.3%)
**Pediatric Volume**		
Low: <1,800 pediatric patients (average of 5 or fewer a day)	1629 (39.3%)	1806 (50.8%)
Medium: 1,800 – 4,999 pediatric patients (average of 6-13 a day)	1248 (30.1%)	1103 (31.0%)
Medium to High: 5,000 – 9,999 pediatric patients (average of 14-26 a day)	708 (17.1%)	367 (10.3%)
High: >=10,000 pediatric patients (average of 27 or more a day)	561 (13.5%)	281 (7.9%)

[a] In 2021, 2 hospitals did not report.
[b] The inpatient services question was asked differently between the two assessments. In 2013, the question was worded, "Which of the following inpatient services may admit children?" In 2021, the question was worded as follows, "Which of the following inpatient services does your hospital have on-site?" The admission of children to an Adult Intensive Care Unit or an Adult Ward/Unit was asked as separate questions in 2021 which offers the best comparison for those data points. Responses from some hospitals were not reported (PDF respondents).
[c] The Physician certification/training question was asked differently between the two assessments. In 2013, the question was worded, "Thinking of the physicians who currently staff your ED and care for children, what types of training are represented?" The question in 2021 was worded, "What types of training/certification are required for physicians who staff your ED 24/7 and care for children?" Responses from some hospitals were not reported (PDF respondents).

© 2023 Remick KE et al. *JAMA Network Open*.

eTable 4. Comparison of Domain Scores by Pediatric Volume Categories: 2013-2021 NPRP Assessments

	Pediatric Patient Volume Category[a]				
	**Low (N = 1423)**	**Medium (N = 864)**	**Medium High (N = 305)**	**High (N = 233)**	**Overall (N = 2825)**
**WPRS 2021**: Median [Q1, Q3]	65.7 [58.6, 78.0]	72.7 [63.6, 89.0]	80.7 [67.2, 95.9]	96.6 [84.9, 100.0]	70.5 [61.3, 87.4]
**WPRS 2013**: Median [Q1, Q3]	67.3 [56.1, 80.3]	74.9 [62.0, 88.4]	79.8 [66.5, 93.1]	96.0 [88.4, 100.0]	72.7 [60.1, 87.8]
**Administration and Coordination domain score 2021**: Median [Q1, Q3]	0.0 [0.0, 9.5]	0.0 [0.0, 19.0]	9.5 [0.0, 19.0]	19.0 [9.5, 19.0]	0.0 [0.0, 19.0]
**Administration and Coordination domain score 2013**: Median [Q1, Q3]	9.5 [0.0, 19.0]	9.5 [0.0, 19.0]	9.5 [0.0, 19.0]	19.0 [19.0, 19.0]	9.5 [0.0, 19.0]
**Personnel domain score 2021**: Median [Q1, Q3]	10.0 [5.0, 10.0]	10.0 [5.0, 10.0]	10.0 [5.0, 10.0]	10.0 [10.0, 10.0]	10.0 [5.0, 10.0]
**Personnel domain score 2013**: Median [Q1, Q3]	5.0 [0.0, 5.0]	5.0 [5.0, 10.0]	5.0 [5.0, 10.0]	10.0 [5.0, 10.0]	5.0 [0.0, 10.0]
**Quality Improvement domain score 2021**: Median [Q1, Q3]	0.0 [0.0, 5.6]	1.4 [0.0, 7.0]	4.2 [0.0, 7.0]	7.0 [7.0, 7.0]	0.0 [0.0, 7.0]
**Quality Improvement domain score 2013**: Median [Q1, Q3]	0.0 [0.0, 4.2]	0.0 [0.0, 7.0]	4.2 [0.0, 7.0]	7.0 [5.6, 7.0]	0.0 [0.0, 5.6]
**Patient Safety domain score 2021**: Median [Q1, Q3]	14.0 [12.1, 14.0]	14.0 [14.0, 14.0]	14.0 [14.0, 14.0]	14.0 [14.0, 14.0]	14.0 [12.1, 14.0]
**Patient Safety domain score 2013**: Median [Q1, Q3]	11.5 [8.3, 14.0]	14.0 [10.2, 14.0]	14.0 [10.2, 14.0]	14.0 [12.1, 14.0]	12.1 [10.2, 14.0]
**Policies and Procedures domain score 2021**: Median [Q1, Q3]	11.8 [7.4, 14.8]	14.0 [9.6, 17.0]	14.8 [10.3, 17.0]	17.0 [14.0, 17.0]	12.6 [8.9, 17.0]
**Policies and Procedures domain score 2013**: Median [Q1, Q3]	9.6 [6.7, 14.0]	11.9 [8.9, 14.8]	12.6 [9.6, 17.0]	14.8 [12.6, 17.0]	11.8 [7.4, 14.8]
**Equipment and Supplies domain score 2021**: Median [Q1, Q3]	32.4 [30.0, 33.0]	33.0 [31.8, 33.0]	33.0 [31.8, 33.0]	33.0 [33.0, 33.0]	33.0 [31.3, 33.0]
**Equipment and Supplies domain score 2013**: Median [Q1, Q3]	31.8 [29.4, 32.5]	32.4 [31.3, 33.0]	32.5 [31.8, 33.0]	33.0 [32.5, 33.0]	32.4 [30.1, 33.0]

[a] Volume totals are based on reported 2021 pediatric volume. Some EDs volume changed from 2013 to 2021

© 2023 Remick KE et al. *JAMA Network Open*.
