# Supplementary material for: National Assessment of Pediatric Readiness of US Emergency Departments During the COVID-19 Pandemic
Source: JAMA Netw Open. 2023 Jul 7;6(7):e2321707. doi: 10.1001/jamanetworkopen.2023.21707 (PMC10329204; doi:10.1001/jamanetworkopen.2023.21707)
Supplement: Supplement 2. — Data Sharing Statement [file jamanetwopen-e2321707-s002.pdf]

Data Sharing Statement

Remick. National Assessment of Pediatric Readiness of US Emergency Departments During the COVID-19 Pandemic. *JAMA Netw Open.* Published July 07, 2023.
doi:10.1001/jamanetworkopen.2023.21707

Data
Data available: Yes
Data types: Data (not involving human participants)
How to access data: pedsready@hsc.utah.edu
When available: With publication

Supporting Documents
Document types: None

Additional Information
Who can access the data: Researchers whose proposed use of the data is approved
Types of analyses: For projects that evaluate impact of pediatric readiness or its components on health systems, hospitals, emergency departments or patient outcomes.
Mechanisms of data availability: After approval of a proposal.
